# An integrated multi-omic analysis of iPSC-derived motor neurons from C9ORF72 ALS patients

**DOI:** 10.1016/j.isci.2021.103221

**Published:** 2021-10-12

**Authors:** The NeuroLINCS Consortium, Hemali Phatnani, Justin Kwan, Dhruv Sareen, James R. Broach, Zachary Simmons, Ximena Arcila-Londono, Edward B. Lee, Vivianna M. Van Deerlin, Neil A. Shneider, Ernest Fraenkel, Lyle W. Ostrow, Frank Baas, Noah Zaitlen, James D. Berry, Andrea Malaspina, Pietro Fratta, Gregory A. Cox, Leslie M. Thompson, Steve Finkbeiner, Efthimios Dardiotis, Timothy M. Miller, Siddharthan Chandran, Suvankar Pal, Eran Hornstein, Daniel J. MacGowan, Terry Heiman-Patterson, Molly G. Hammell, Nikolaos.A. Patsopoulos, Oleg Butovsky, Joshua Dubnau, Avindra Nath, Robert Bowser, Matt Harms, Mary Poss, Jennifer Phillips-Cremins, John Crary, Nazem Atassi, Dale J. Lange, Darius J. Adams, Leonidas Stefanis, Marc Gotkine, Robert H. Baloh, Suma Babu, Towfique Raj, Sabrina Paganoni, Ophir Shalem, Colin Smith, Bin Zhang, Brent Harris, Iris Broce, Vivian Drory, John Ravits, Corey McMillan, Vilas Menon, Lani Wu, Steven Altschuler, Jonathan Li, Ryan G. Lim, Julia A. Kaye, Victoria Dardov, Alyssa N. Coyne, Jie Wu, Pamela Milani, Andrew Cheng, Terri G. Thompson, Loren Ornelas, Aaron Frank, Miriam Adam, Maria G. Banuelos, Malcolm Casale, Veerle Cox, Renan Escalante-Chong, J. Gavin Daigle, Emilda Gomez, Lindsey Hayes, Ronald Holewenski, Susan Lei, Alex Lenail, Leandro Lima, Berhan Mandefro, Andrea Matlock, Lindsay Panther, Natasha Leanna Patel-Murray, Jacqueline Pham, Divya Ramamoorthy, Karen Sachs, Brandon Shelley, Jennifer Stocksdale, Hannah Trost, Mark Wilhelm, Vidya Venkatraman, Brook T. Wassie, Stacia Wyman, Stephanie Yang, NYGC ALS Consortium, Jennifer E. Van Eyk, Thomas E. Lloyd, Steven Finkbeiner, Ernest Fraenkel, Jeffrey D. Rothstein, Dhruv Sareen, Clive N. Svendsen, Leslie M. Thompson

**Affiliations:** 1 Department of Biological Engineering, Massachusetts Institute of Technology, Cambridge, MA 02139, USA; 2 UCI MIND, University of California, Irvine, CA 92697, USA; 3 Department of Biological Chemistry, University of California, Irvine, CA 92697, USA; 4 Department of Neurobiology and Behavior, University of California, Irvine, CA 92697, USA; 5 Department of Psychiatry and Human Behavior, University of California, Irvine, CA 92697, USA; 6 Sue and Bill Gross Stem Cell Center, University of California, Irvine, CA 92697, USA; 7 Center for Systems and Therapeutics and the Taube/Koret Center for Neurodegenerative Disease, Gladstone Institutes, University of California, San Francisco, San Francisco, CA 94158, USA; 8 Departments of Neurology and Physiology, University of California, San Francisco, San Francisco, CA 94158, USA; 9 Brain Science Institute, Johns Hopkins University School of Medicine, Baltimore, MA 212056, USA; 10 Department of Neurology and Neuroscience, Johns Hopkins University School of Medicine, Baltimore, MA 212056, USA; 11 Cellular and Molecular Medicine Program, Johns Hopkins University School of Medicine, Baltimore, MA 212056, USA; 12 The Board of Governors Regenerative Medicine Institute, Cedars-Sinai Medical Center, 8700 Beverly Boulevard, Los Angeles, CA 90048, USA; 13 Advanced Clinical Biosystems Research Institute, The Barbra Streisand Heart Center, The Smidt Heart Institute, Cedars-Sinai Medical Center, Los Angeles, CA, USA

**Keywords:** Biological sciences, Neuroscience, Systems neuroscience, Systems biology, Omics

## Abstract

Neurodegenerative diseases are challenging for systems biology because of the lack of reliable animal models or patient samples at early disease stages. Induced pluripotent stem cells (iPSCs) could address these challenges. We investigated DNA, RNA, epigenetics, and proteins in iPSC-derived motor neurons from patients with ALS carrying hexanucleotide expansions in *C9ORF72*. Using integrative computational methods combining all omics datasets, we identified novel and known dysregulated pathways. We used a *C9ORF72* Drosophila model to distinguish pathways contributing to disease phenotypes from compensatory ones and confirmed alterations in some pathways in postmortem spinal cord tissue of patients with ALS. A different differentiation protocol was used to derive a separate set of *C9ORF72* and control motor neurons. Many individual -omics differed by protocol, but some core dysregulated pathways were consistent. This strategy of analyzing patient-specific neurons provides disease-related outcomes with small numbers of heterogeneous lines and reduces variation from single-omics to elucidate network-based signatures.

## Highlights

• Multi-omic analysis of differentiated C9ORF72 iPSC-derived motor neurons
• Network-based integrative computational analysis
• Pathogenic versus compensatory pathways elucidated using C9ORF72 *Drosophila* model
• Pathways confirmed with alternative differentiation protocol and postmortem data

## Graphical abstract

**Figure:**
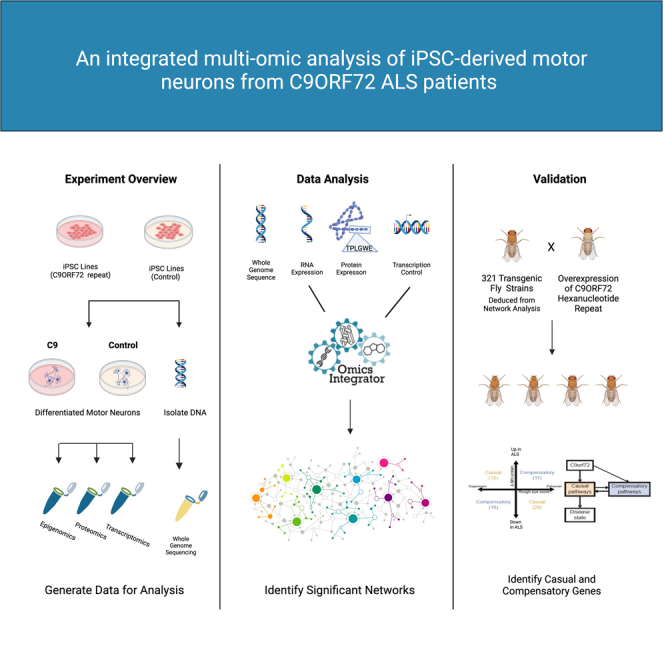

## Introduction

Modeling neurological diseases using induced pluripotent stem cell (iPSC) technology offers a unique platform to study the process of pathogenesis. Rather than using artificially expressed human disease genes in mice or end-stage postmortem tissues from patients, the generation of new neurons and astrocytes from patient-specific cells allows for discovery of the earliest genesis of disease signatures. One neurodegenerative disease group that has been modeled extensively using iPSCs is the motor neuron disorders. Adult-onset motor neuron diseases include amyotrophic lateral sclerosis (ALS), where motor neurons degenerate late in life, inevitably leading to paralysis and asphyxiation. Genetic underpinnings have been identified in ∼15% of ALS cases (Paez-Colasante et al., 2015). Of these, the most common mutation is a hexanucleotide repeat expansion (HRE) in the first intronic region of *C9ORF72,* which accounts for over 40% of all known familial and 10% of known sporadic forms of the disease. In healthy individuals, fewer than 24 copies of the GGGGCC HRE are present within the first intron of the *C9ORF72* gene. However, in disease this GGGGCC sequence is expanded hundreds to thousands of times. Although there is no known correlation between HRE length and disease severity, this intronic expansion leads to three pathologic hallmarks of C9ORF72 ALS/frontal temporal dementia (FTD). First, the HRE has been shown to lead to reduced C9ORF72 RNA and protein expression, leading to a loss of function of the *C9ORF72* gene. Second, there is a gain of toxic function via the bidirectional transcription of the GGGGCC HRE leading to the production of toxic G_4_C_2_ and G_2_C_4_ repeat RNA species, which are thought to sequester and impair the function of RNA-binding proteins. Third, there is a gain of toxic function via the non-canonical RAN translation of repeat RNAs to produce five toxic dipeptide repeat proteins (Poly(GR), Poly(GA), Poly(GP), Poly(PR), and Poly(PA)), which are proposed to impair multiple cellular processes (Swinnen et al., 2020; Jiang and Ravits, 2019; Balendra and Isaacs, 2018). Although much is known about the mutation and abnormal proteins that are produced by its transcripts (Brown and Al-Chalabi, 2017), it still remains unclear as to how repeats in *C9ORF72* lead ultimately to neuronal dysfunction and death.

Some of the first disease modeling studies showed that iPSCs could be generated from early-onset motor neuron diseases such as spinal muscular atrophy and that these motor neurons exhibited disease-specific cell death *in vitro* (Ebert et al., 2009; Fuller et al., 2015; Ng et al., 2015; Nizzardo et al., 2015; Sareen et al., 2012; Vazquez-Arango et al., 2016). Of interest, for later-onset motor neuron diseases such as ALS, initial studies with iPSC models did not show overt death in motor neurons (Dimos et al., 2008). However, for inherited forms of ALS such as *C9ORF72* repeat expansions (C9) there were specific changes in neuronal activity, gene expression, and cellular processes (Devlin et al., 2015; Donnelly et al., 2013; Sareen et al., 2013; Selvaraj et al., 2018; Shi et al., 2018; Wainger et al., 2014). More recently, stressors, such as trophic factor withdrawal, have led to cell death phenotypes (Shi et al., 2018), although it is not clear how these stressors relate to human disease onset and progression. Subsets of sporadic patients with ALS also showed phenotypic changes including reduced fiber outgrowth at later time points in culture (Fujimori et al., 2018), although a comprehensive -omics analysis was not performed and *C9ORF72* cases were not included. These iPSC models provide a unique opportunity to examine the molecular changes that occur due to ALS-causing genes in motor neurons. Postmortem studies (Delic et al., 2018; Emde et al., 2015; Pare et al., 2018; Prudencio et al., 2015; Sanfilippo et al., 2017) have provided important insights into these processes, however patient samples often represent a late stage of the disease with extensive degeneration, which may not exhibit molecular or cellular signatures directly associated with the initiating events that cause the disease. By contrast, neurons derived from iPSCs can provide insights into the earliest stages of neurodegeneration, opening a window into the period when therapeutics might have the greatest benefit.

Although iPSCs provide critical models that represent disease within their human context, it is clear that one of the major challenges for the field is patient-to-patient variation in iPSC lines and major challenges regarding the reliable and reproducible differentiation of motor neurons from iPSCs. Batch-to-batch variation in differentiations are driven by factors difficult to control, such as slight variances in the multitude of small molecules and other media components along with plating densities and technical variations in feeding and handling. The combined heterogeneity in iPSC state and differentiation protocols means that even within the same laboratory it is difficult to control completely and that comparing data between laboratories using different protocols is almost impossible.

The goal of the current study was to use a multi-omic approach to investigate whether a network-based analysis would facilitate identification of early pathogenic events in C9-ALS where the signal was strong enough to rise above the noise of the system. We differentiated cells for all the assays at once and then divided the cells for each -omics, using stringent quality control measures in both experimental and analytical steps. Analysis of the primary assays was performed. We also developed an integrative approach that combines multi-omic data using network-based algorithms. Significant signals emerged even with a small sample size. We then carried out experiments in a *Drosophila* model to test whether the “hits” were relevant to C9-ALS and if they were pathological changes or compensatory responses to neurodegeneration, and we integrated these data into a map of pathway changes in C9-ALS patient cells.

A critical test of this approach is whether similar findings can be found in cells from different donors or differentiation states. To that end, we differentiated a different set of lines using a different differentiation protocol, but applied the same integrative approach. Despite the many experimental differences leading to non-overlapping changes in specific genes and proteins, our computational approach confirmed changes that were detected in both sets of samples at the network level. These studies support the feasibility of a network-based multi-omic approach to generate disease-related hypotheses. More patients will now be required to investigate and validate these pathways further in continuing experiments. As part of the NIH-funded NeuroLINCS consortium, all of the datasets along with the data integration have been posted to a portal for data sharing of this unique resource https://lincsproject.org and the iPSC lines are all available at https://stemcells.nindsgenetics.org/. This study has led to the formation of Answer ALS where 1,000 iPSC lines are currently being generated and analyzed using similar integrative analyses in familial and sporadic patients to explore the power of this approach to further stratify ALS subpopulations and identify therapeutic targets.

## Results

### Generation and characterization of iPSC lines

**Figure 1:**
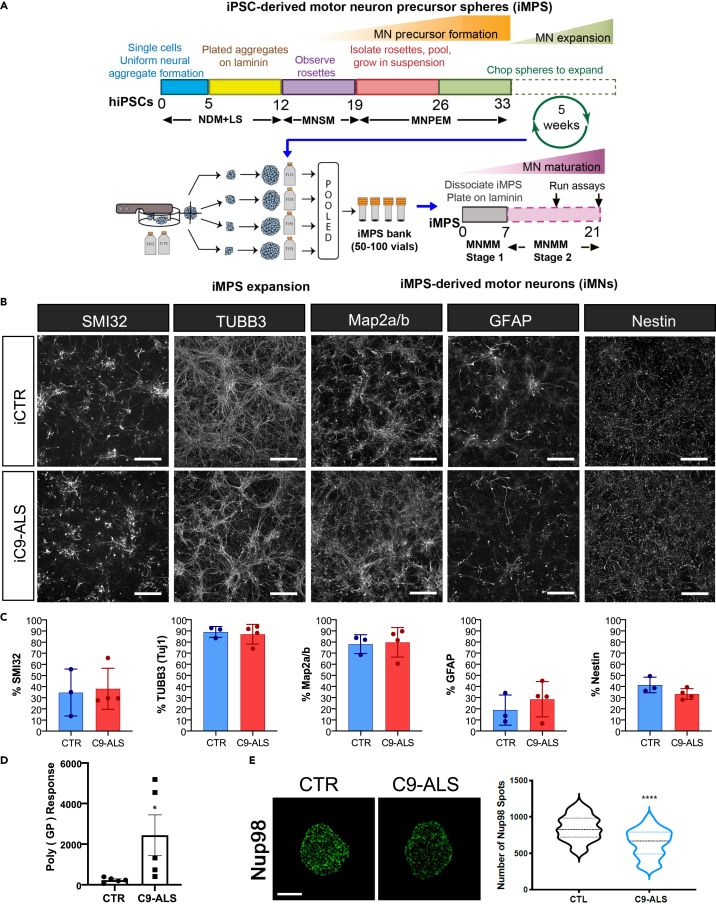
iPSC differentiations (A) Schematic of protocol for iPSC differentiation into motor neuron cultures used by NeuroLINCS for transcriptomics, proteomics, and epigenomics assays. The iPSC-derived motor neuron precursor spheres (iMPS) were dissociated into single cells from C9-ALS and healthy patient iPSC lines and plated on laminin substrate to differentiate further into motor neuron (iMN) cultures over 21 days. (B) Representative images of iMNs from control (25iCTR) and C9-ALS (52iALS). iMNs show consistent distribution of neural cell populations marked by SMI32, TuJ1, Map2a/b, GFAP, and nestin. Scale bars are 50 μm. (C) Levels of SMI32, TUBB3 (TuJ1), GFAP, nestin, and Map2a/b in control and C9-ALS iMN cultures from the individual iPSC lines. Two-sided unpaired t test with Welch's correction (CTR n = 3 and C9-ALS n = 4). (D) Poly(GP) DPR levels as determined by MSD ELISA assay in iMNs (from CS29 ISO 191.06, CS52 4544.25, CS0702 60.45, CS7VCZ 5180.33, CS29 405.69, CS0465 297.85, CS0594 391.5, CS0BUU 1323.32, CS52 ISO 233.72, CS6ZLD 738.54). p = 0.0348. (E) Maximum intensity projections from SIM imaging of Nup98 in nuclei isolated from control and C9ORF72 iMNs (CS0188, CS0594, CS0702, CS29, CS52, CS7VCZ). Quantification of Nup98 spots. N = 3 control and 3 C9ORF72 iPSC lines, 20 NeuN+ nuclei/line. Student’s t test was used to calculate statistical significance (Gendron et al., 2017). p < 0.0001. Scale bar, 5 μm.

An initial set of four C9-ALS lines and three control iPSC lines generated from patient fibroblasts and reported on previously (Sareen et al., 2013) were used for the majority of this study. We have previously shown that motor neurons derived from these C9-ALS lines exhibited RNA foci, physiological changes that include a diminished capacity to fire continuous spikes and changes in specific genes (Sareen et al., 2013). Furthermore, we extended the number of lines to an additional seven controls and six C9-ALS iPSC lines but using peripheral blood mononuclear cells (PBMCs) as the starting cell source for a replication cohort. All lines were generated using episomal plasmid-based reprogramming methods. iPSC lines (list in Figures S6 and S7) were differentiated first to motor neuron precursor spheres (iMPS) that were then further differentiated into motor neurons (iMNs) as described in STAR Methods, with no overall differences in cell markers (Figures 1A–1C). The lines retained their repeat expansion mutation following reprogramming as described previously (Ho et al., 2020; Sareen et al., 2013). All iPSC lines maintained normal karyotypes as determined by G-band karyotyping (Figures S4A and S4B), and the identity of iPSCs and differentiated iMNs was confirmed to match the parent fibroblasts or isolated PBMCs by DNA fingerprinting (Figures S6 and S7).

We also evaluated C9 phenotypic signatures in subsets of the lines to determine that the iMNs produced relevant C9ORF72 pathology. We evaluated dipeptide repeat (DPR) species using immunoassays to evaluate the expression of Poly(GP) as described (Gendron et al., 2017). In accordance with previous reports, Poly(GP) production is highly variable in individual patient lines (Gendron et al., 2017); however, compared with controls, C9-ALS iMNs produce significantly more Poly(GP) (Figure 1D). Impaired nucleocytoplasmic transport and alterations in the expression and localization of specific nucleoporins that comprise the nuclear pore complex to govern functional nucleocytoplasmic transport have emerged as a prominent pathologic hallmark of multiple neurodegenerative diseases including C9ORF72 ALS/FTD. To verify that nucleoporin components are altered, we performed super resolution structured illumination microscopy (SIM) on nuclei isolated from control and C9-ALS iMNs immunostained for Nup98 and quantified as previously described (Coyne et al., 2020b; Gendron et al., 2017). Nuclear preparations and SIM are required to identify changes in nuclear pore proteins, which are not readily observed through proteomic analysis. In comparison with controls, we observed a significant reduction in the nuclear expression and localization of Nup98 in C9-ALS iMNs (Figure 1E) similar to previous pathological observations in iPSCs and postmortem tissue (Coyne et al., 2020b).

### Whole-genome sequencing shows no known disease-associated ALS variants

Whole-genome sequencing (WGS) was performed on the initial set of C9-ALS and control iPSC lines in order to establish methodologies and provide a reporting of variants in disease-modifying genes to help elucidate and interpret line-to-line variability, despite the fact that the majority of these variants are benign or of unknown significance. A novel computational pipeline was used to annotate the variants in the genomes of the control and C9-ALS lines relative to reference human genomes (see Whole genome sequencing and analysis methods). The number of single nucleotide polymorphisms (SNPs) was within the expected range, and there were no overt genetic abnormalities. Across all lines, we found 11,260,464 variants with 9,197,462 variants in the control lines and 8,818,235 variants in the C9-ALS lines. Thus, there was an average of 5.4 million variants per line, which is consistent with the variation that has been observed in human genomes (Auton et al., 2015). After applying annotation (see STAR Methods), we filtered for exonic functional variation (Table S6). There were 57,910 exonic functional variants in the controls, and 12,898 were rare (less than 1%) or novel (no frequency information). There were 55,815 exonic functional variants in the C9-ALS lines, and 8,225 were rare or novel (Table S6). Next, we investigated if any of the lines had genetic variants previously associated with ALS, and we found three variants in OPTN, ALS2, and DIAPH3 that have been associated with ALS but are also found at relatively high frequency (>2%) in the general population (Table S7). Other variants in ALS-associated genes were observed, but none that were known previously to be disease associated or causing. However, of interest, the 52i ALS line contains the APOE-ε4 allele (rs429358) (C130R), which is associated with an increased risk of developing Alzheimer’s disease (Farrer et al., 1997). We next applied the American College of Medical Genetics gene criteria to identify pathogenic or likely pathogenic variants (Table S8). Although a subset of these variants is in ALS genes that are listed in the ASLoD database (Wroe et al., 2008), to our knowledge none of these variants are expected to confer risk of developing ALS. WGS analysis of the patient cell lines revealed no pathogenic or likely pathogenic variants; hence, there is no indication that these variants confer risk or influence risk of developing ALS.

### Transcriptomic analysis of C9-ALS versus control iMNs

**Figure 2:**
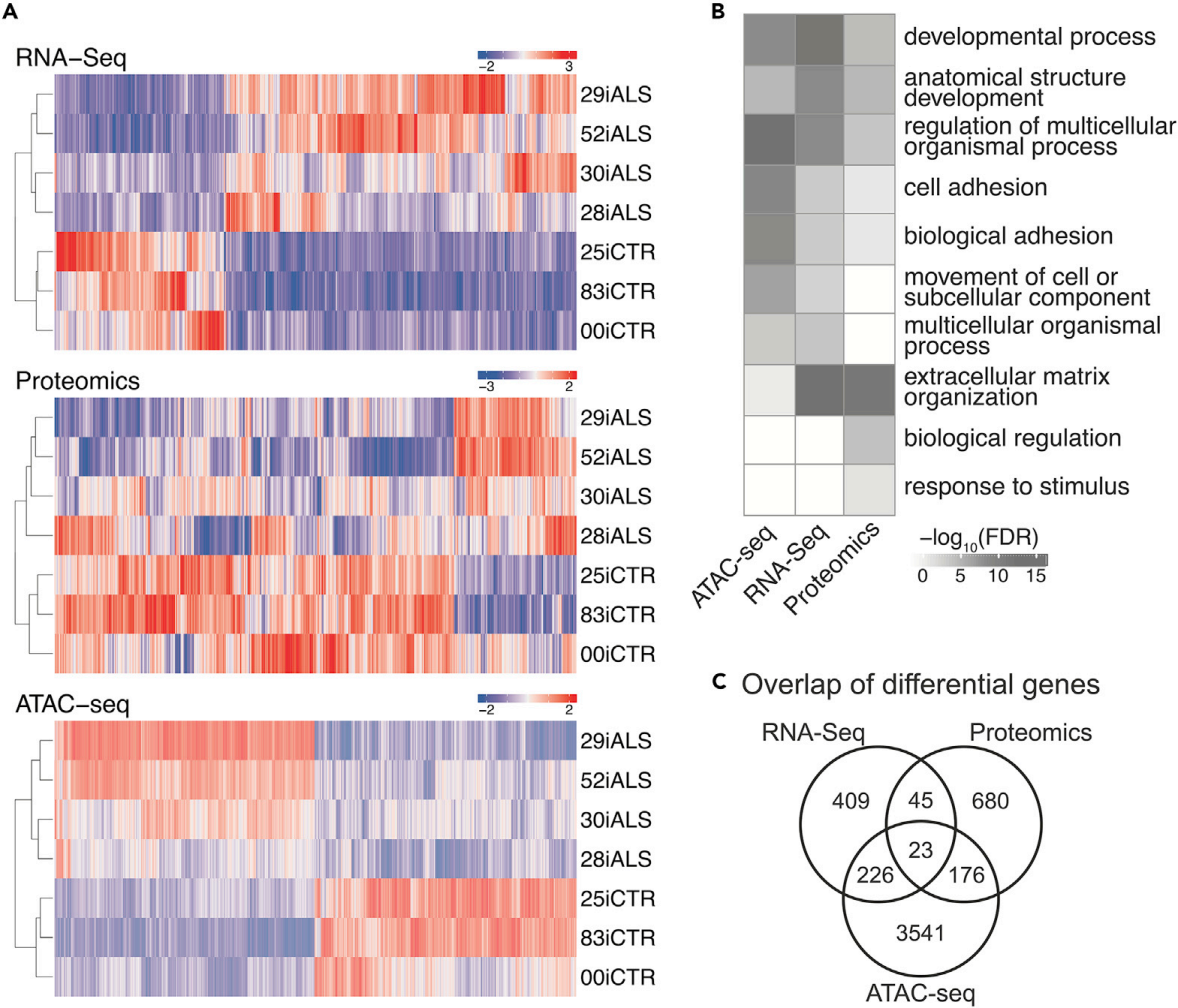
OMIC assays (A) Hierarchical clustering of RNA-Seq, Proteomics, and ATAC-seq signals normalized by *Z* score. (B) Top GO term enrichments for each assay reveal common biological processes. The top five GO process terms (sorted by FDR significance) for each assay were included in the visualization. (C) Venn diagram of differential genes or proteins from each assay. Each differential ATAC-seq peak was assigned the nearest protein coding gene (up to a limit of 50 kb from the TSS).

To identify the earliest molecular changes in the differentiated ALS iMNs, we carried out parallel multi-omics analyses. RNA sequencing revealed transcriptomic signatures associated with the C9-ALS lines (Table S9). Total RNA-Seq (Ribo-Zero rRNA depletion) was carried out on the distributed iMN pellets as described in STAR Methods with principal component analysis (PCA) shown in Figure S8A. Statistical analysis of differential expression was performed using DEseq2. We found 828 differentially expressed transcripts (271 downregulated and 557 upregulated) between C9-ALS and control iMNs (false discovery rate [FDR]<0.1), of which 704 were annotated as protein-coding in Uniprot (Table S9; Figures S9A and S9B). Of these 828 differentially expressed genes (DEGs), *C9ORF72* was not differentially expressed. Exploratory analysis of gene expression levels was carried out using hierarchical clustering (Figure 2A). To begin to understand the effect of the C9 mutation on a multicellular culture, genes that were significantly different between C9-ALS and control samples were used for Cell Type-Specific Expression Analysis (CSEA; Xu et al., 2014) (Figure S9C). CSEA revealed an enrichment of cortical- and motor neuron-specific gene expression. Next, Gene Ontology (GO) analysis was conducted to determine the functional role of these genes using GOrilla on the 704 DEGs revealing an enrichment in extracellular matrix (ECM) and cell adhesion terms, which included ECM disassembly, ECM organization, collagen binding, and focal adhesion (Figures 2B, S9D, and S9E).

To identify potential upstream regulators controlling the differential expression of these ECM-related genes, Ingenuity Pathway Analysis (IPA) was conducted. Some of the top predicted regulators identified include SMADs (transforming growth factor β [TGFβ] signaling), mitogen-activated protein kinase 1 (ERK), and nuclear factor kappa B (NF-κB). Network-based analysis of upstream regulators and gene targets showed a TGFβ, AP-1 transcription factor subunit (AP1), erb-b2 receptor tyrosine kinase 2 (ERBB2), plasminogen activator, urokinase receptor (PLAUR), and neuregulin 1 (NRG1) network that were predicted to regulate many of the ECM and cell adhesion-related DEGs (Figure S9D). Of note, NRG1 was identified as a major hub gene that could regulate other upstream regulators and directly regulate *ACTIN* and *INTEGRIN* expression, each of which was upregulated in the ALS iMNs. Matrix metalloproteinases (*MMP*s) were significantly dysregulated, in all cases showing increased expression, and were downstream of the NRG1 hub (Figure S9E). We further investigated dysregulation of these *MMPs* and found that their corresponding substrates (e.g., *LAMININs*, *COLLAGENs*) were also upregulated (Figure S9E). These data indicate a possible role for NRG1 in the dysregulation of ECM and cell adhesion-related genes in ALS iMNs, as suggested previously in mouse models of ALS (Song et al., 2012).

The use of deep RNA-Seq allowed further analysis of the transcriptomic data focused on alternative splicing. This analysis was conducted using rMATS (Shen et al., 2014). Figure S9F shows the percentage of significant alternative splicing events found in the ALS iMNs compared with controls, showing a high percentage of Exon skipping (ES, 57%) and Intron retention (RI, 26%). This same pattern was previously identified as enriched in studies using human familial ALS and sporadic ALS patient tissue (Prudencio et al., 2015). GO enrichment analysis of these alternatively spliced genes revealed some similar terms found in the gene level differential analysis like cell adhesion, and also unique terms related to RNA processing, axonal guidance, and translation (Figures S9G–S9I).

A previous study (Sareen et al., 2013) using the same C9-ALS iPSC-derived motor neurons and 2 of the same controls, but a very different differentiation protocol, also showed dysregulation; in this case 66 genes between 4 ALS and 4 control samples with a fold change of >2 and p value <0.05 were dysregulated. Of those 66 genes, 8 genes overlapped with the 828 DEGs from our study, although in different directions. Although specific genes differed, even with this small number of overlapping genes from different studies using distinct differentiation protocols and different RNA-Seq platforms, GO enrichment analysis revealed an enrichment for extracellular region in the 66 genes, similar to our analysis. This suggests that batch, differentiation, and study effects make direct comparisons difficult at the individual gene level but disease overlapping signatures remain at the global pathway levels. This challenge formed the rationale to include proteomics and ATAC-seq and execute large network-based analyses as described in the data integration below, with the hypothesis that signatures that separate ALS from control at the pathway and network levels would enable discovery of mechanistic disease-relevant pathways.

### Proteomics show ECM and mRNA processing dominate protein changes

A sample-specific library using DDA-based acquisition files was compiled and DIA-MS samples were run against the peptide library. Data quality was assessed by MS1 and MS2 total ion current, normalized protein intensity distribution, number of unique and shared hits identified, and correlation between ALS and control lines (Figures S10A–S10D). Using this method, we were able to identify 3,844 unambiguous proteins based on 23,436 unique peptides (Figure S10A). MAP DIA software was then used to determine relative peptide and protein amounts within the samples, as well as log2FC between C9-ALS and control using transition level data (Teo et al., 2015). Using a 1% FDR, 95% confidence interval, and 0.6 abs(log2FC) cutoff, a final list of 924 differentially expressed proteins (DEPs) was obtained, which did not include C9ORF72. Hierarchical clustering of differential protein intensity values showed similar groupings between biological replicates for ALS and control samples as seen for RNA-Seq and ATAC-seq (Figure 2A). Of interest, unbiased analysis of all measured proteins resulted in separation between control and ALS groups (Figure S10F).

A small subset of the DEPs (6.8%) had overlap with both the ATAC-seq and RNA-Seq (Figure 2C), specifically, 68 common DEGs/DEPs (45 between RNA and protein and 23 between all -omics datasets). mRNA levels do not always predict protein abundance owing to differences in protein turnover (e.g., Stewart et al., 2019 and references therein), further highlighting the importance of incorporating integrated analytical approaches. The fold change values of these overlapping terms have a Spearman correlation R^2^ = 0.76, suggesting that most of the differentially expressed terms that are common have concordant fold change values and directionality (Figure S10E). Of these common proteins, downregulated proteins (13) did not yield any GO enrichment terms (Table S10b). Common upregulated proteins/genes (55) show enrichment in extracellular matrix terms (Table S10a).

856 DEPs did not overlap directly with DEGs. Of these, 183 proteins were upregulated and enriched for extracellular matrix proteins (Table S10c), similar to the enrichment observed for transcriptomic analysis. In addition, network-based analysis of all DEPs (924) by IPA revealed predicted upstream regulators, including TGFβ and SMAD4 (Table S10e), which in turn regulate many of the extracellular matrix genes and proteins identified in the differential RNA and protein analysis, respectively, and integrated -omics described below.

The remaining unique subset of proteins (674 DEPs) were downregulated and showed enrichment for poly(A) RNA binding, RNA binding, and RNA/mRNA splicing (Table S10d). In addition, IPA of the differential proteins (924) shows predicted inhibition of RNA/mRNA splicing based on downregulation of proteins associated with this pathway (Figure S10G; Table S10f–g). Finally, proteins associated with alternative splicing of mRNA are dysregulated, with most of these proteins decreasing in the ALS neurons. Taken together, this could imply that these downregulated proteins are associated with the altered exon usage and alternative splicing in ALS found in the transcriptomic analysis.

### ATAC-seq shows epigenetic changes due to C9 expression

We sought to study the accessible chromatin landscape in C9-ALS patients and controls. The density of transposase Tn5 cleavage fragments provides a continuous measurement of chromatin accessibility via ATAC-seq (Figure S11A). Analysis of the open chromatin data identified 128,299 peaks that were active in two or more ALS or control samples. Approximately 14% (18,407) accessible regions localize to gene promoters as defined by GENCODE (Harrow et al., 2012); 27% (34,543) lie within 2.5 kb of a transcription start site (TSS). Nearly half of the peaks lie in intronic regions, whereas about a third lie between genes (Figure S11B).

To study alterations in chromatin accessibility in the disease state, we identified and characterized peaks with significantly changed accessibility between C9-ALS and control samples. Roughly 12% (15,814 peaks; FDR < 0.1) of all peaks were found to be differentially open, of which approximately half (7,937) were less accessible in C9-ALS samples (Figure S11C). Hierarchical clustering of differentially open regions revealed similar groupings of patient samples as in RNA-Seq and proteomics (Figure 2A). Correlation coefficients were 0.46 for RNA and ATAC and 0.13 for protein and ATAC, with both comparisons indicating the same direction. Differentially accessible peaks were biased away from regions near TSSs, with only 5.0% (783) annotated to promoters (Figure S11C). Examples of changing chromatin accessibility in ALS versus control lines can be seen in data files (Figure S11C). Next, we sought to answer whether chromatin changes influence broad categories of genes by assigning each peak to its nearest RefSeq gene TSS within 50 kb. A total of 2,345 genes were associated with more ALS peaks than control and were enriched for signaling and calcium ion binding GO terms. Conversely, 2,617 genes were associated with more control peaks than ALS and were enriched for terms such as neuron development and axon guidance (Figure S11D). Overall, ATAC-seq identified many regulatory changes that were consistently different across ALS and control lines. In the data integration section, we analyzed how these changes correspond to changes in RNA-Seq to understand differences in gene regulation between disease and control states.

### Whole-genome analysis and RNA-Seq data integration to identify potential expression quantitative trait loci

Our analysis of the control and ALS lines revealed genomic variants in loci other than the C9ORF72 locus that could potentially contribute to the line-specific differences in the RNA-Seq and proteomic data and influence data integration outcomes. Therefore, we evaluated whether any of the genetic coding variants outside the C9ORF72 locus were disproportionately present in C9-ALS lines compared with controls in order to determine whether specific genetic variants might drive expression differences between C9ORF72 and control lines that are not directly regulated by the disease mutation and could potentially confound the interpretation. Although this is a very small dataset and underpowered to draw significant conclusions, the goal was to establish a method and example to evaluate variants that may alter or confound the identification of signatures specifically attributable to ALS-associated HRE in C9ORF72. For example, we observed that a missense mutation in exon 17 of the poly(ADP-ribose) polymerase 1 (*PARP1*) gene (V762A) was present in all four C9-ALS lines but present in only one of the controls (Figure S12). As this was one of the genes found in the nodes of the integrated network (see below), it is possible that changes observed in the RNA-Seq data could be due to this genomic variant rather than a consequence of the HRE in C9ORF72. Furthermore, we have no reason to believe that this variant is a haplotype that is associated with the C9ORF72 expansion. Therefore, we sought to relate the whole-genome analysis (WGA) to the -omics results to better determine which genes were differentially expressed due to the HRE in C9ORF72 and which might be due to line-specific genetic variation at other loci. Although the sample size is too low for expression quantitative trait loci (eQTL) analysis, we performed this analysis to identify potential eQTL that can be reassessed using a larger sample size in future studies. The methodology we used follows standard statistical analysis for eQTL identification. We focused on exonic variants and found 7,235 nonsynonymous variants that were enriched in either control or ALS cases (Table S11). Then, we compared the genes in which these variants were found with the DEGs (FDR < 0.1, which corresponds to p < 0.015) in C9-ALS or control samples by RNA-Seq. We observed 801 variants (including missense, stop gain, start loss, splicing, frameshift) in genes that were differentially expressed (Table S11). To examine if these subsets of DEGs were significantly correlated to the presence of the variant, we performed linear regression. After voom normalization of the gene expression counts, using the limma package, a linear model was fit to each normalized gene expression-variant comparison. Adjusted R^2^ and Benjamini-Hochberg adjusted p values were calculated for each linear fit. This linear regression analysis revealed 69 variants that could be influencing the expression of 56 genes and confounding the identification of C9ORF72 ALS-specific gene expression differences (Table S12). Seven of these genes were found in the final network analysis, but some discordance can be seen in the genotype-expression comparisons (Figure S12), which could be due to the limited number of samples for the regression analysis. To further assess whether genetic variants in our samples were confounding the identification of an ALS signature, we compared the variants that were enriched in either the control or ALS cases to known brain-specific eQTLs from the xQTL database (Ng et al., 2017). There were 73,142 variants in our samples that overlapped with key known brain eQTLs that represented 5,292 genes; of these genes, 114 overlap genes were found to be significantly differentially expressed in the ALS versus control cases. Nineteen of these variants were found in all cases of one group only versus the other group, e.g., all ALS cases and no controls or no ALS case and all controls. These 19 variants are known eQTLs for seven genes that were also found in the RNA-Seq analysis to be differentially expressed between ALS and control groups (Figures S12 and S13), one of which, integrin subunit alpha V (*ITGAV*), was identified as dysregulated in each primary assay, WGA, network, and as a fly modifier gene. Of the genes that had unbalanced genotype variants between ALS and control iPSC samples, only two of these overlapped and showed a significant impact in the fly phenotype screen, PARP1 and CALD1. These analyses demonstrate that the known brain eQTLs are likely to have at most a modest effect on the expression differences between C9ORF72 and control lines in our study and also provide a path for future studies with large patient cohorts.

### RNA-Seq, proteomics, and ATAC-seq data comparison

In order to characterize the similarities and differences between the genomics, RNA-Seq, proteomics, and ATAC-seq experiments, we first examined the overlap of the RNA, protein, and epigenomics assays. Each differentially open region was assigned the nearest protein-coding gene (up to a limit of 50 kb from the TSS). The sets of genes and proteins detected by each assay all showed a modest increase in overlap compared with what would have been expected by chance. For example, approximately 7% of the proteins that differed between ALS and control samples were also differentially expressed in the RNA-Seq data (p value = 1.92 × 10^−14^). A higher fraction of genes that differed in RNA expression also showed changes in ATAC-seq (38%; p value = 1.86 × 10^−14^) and 14% of the proteins that differed between ALS and control samples were also differential ATAC-seq genes (p value = 0.056). All three assays were also enriched for similar biological processes, for instance, comparison of the top GO terms from each experiment were enriched for adhesion and extracellular matrix processes, supporting biological overlap between assays (Figures 2 and S14).

### An “omics integrator” reveals novel C9-specific pathogenic pathways

The challenges in comparing diverse molecular assays requires a more integrated and systems-based approach. Careful integration of the various -omics data with each other and prior knowledge from the literature provide an opportunity to uncover causal relations. For example, a joint analysis of epigenomics and transcriptional data can uncover evidence of activity changes in key transcriptional regulators, which tend to be difficult to detect using mass spectrometry. Similarly, mapping proteomic data onto networks representing protein interactions can reveal functional relations among the DEPs. In order to explore these relationships, we used a strategy implemented in Omics Integrator (Tuncbag et al., 2016), which begins by using motif analysis of open chromatin regions near DEGs to identify differentially active transcription factors (TFs). Omics Integrator then uses network optimization to search a vast database of protein-protein interactions to discover, *de novo*, pathways linking the experimentally determined proteomic data and the inferred TFs.

### Identification of transcriptional regulators

**Figure 3:**
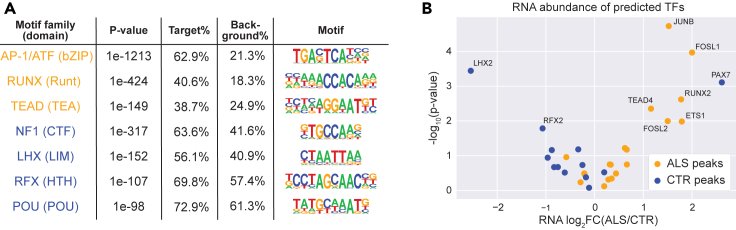
Transcription factor predictions (A) Transcription factor (TF) families that are predicted to be differentially active between ALS and control samples. Orange motifs are predicted to be more active in ALS, and blue motifs are predicted to be more active in controls. (B) A volcano plot of RNA abundance for each predicted TF shows that TFs that are predicted to be active in ALS are also more highly expressed in ALS samples, whereas TFs that are predicted to be active in controls are less expressed in ALS samples.

Potential transcriptional regulators were identified using *de novo* DNA motif analysis as a first step. To capture regulators mediating changes in chromatin accessibility, we searched for motifs that are enriched in differentially accessible peaks. We also searched within peaks that changed in accessibility and were near DEGs to identify transcriptional regulators that drive changes in gene expression. Peaks that were less accessible in C9-ALS samples were enriched for several TFs including Nuclear Factor I (NF1) family that controls the onset of gliogenesis in the developing spinal cord (Deneen et al., 2006) and LIM Homeobox (LHX) TFs that regulate expression of axon guidance receptors (Palmesino et al., 2010) (Figure 3A). Conversely, peaks that were more accessible in C9-ALS samples were enriched for AP-1, RUNX2, and TEAD4. Altered AP-1 activity, which was independently predicted by IPA of the transcriptomics data, has previously been described in the SOD1 mouse model of ALS (Bhinge et al., 2017). Of note, we found that RNA transcripts corresponding to motifs enriched in C9-ALS peaks are upregulated in C9-ALS samples, whereas transcripts corresponding to motifs enriched in control peaks are downregulated in C9-ALS samples (Figure 3B). These results suggest that epigenetic changes could be driven by differences in expression of transcription factor encoding transcripts.

### A network of C9ORF72-induced changes

In the next phase of the integration, we combined the transcriptional regulators inferred from RNA-Seq and ATAC-seq with the proteins detected in mass spectrometry. Our approach sought to discover, *de novo*, the cellular pathways that are differentially active between C9 and control lines. The challenge is to go beyond the limited information available in annotated pathways while still avoiding an uninterpretable network containing thousands of interactions. Our approach searches for previously reported protein-protein interactions that directly or indirectly connect our proteomics and transcriptional regulatory data. The method considers the strength of experimental evidence supporting each reported protein-protein interaction from the database and the strength of evidence supporting our own data.

**Figure 4:**
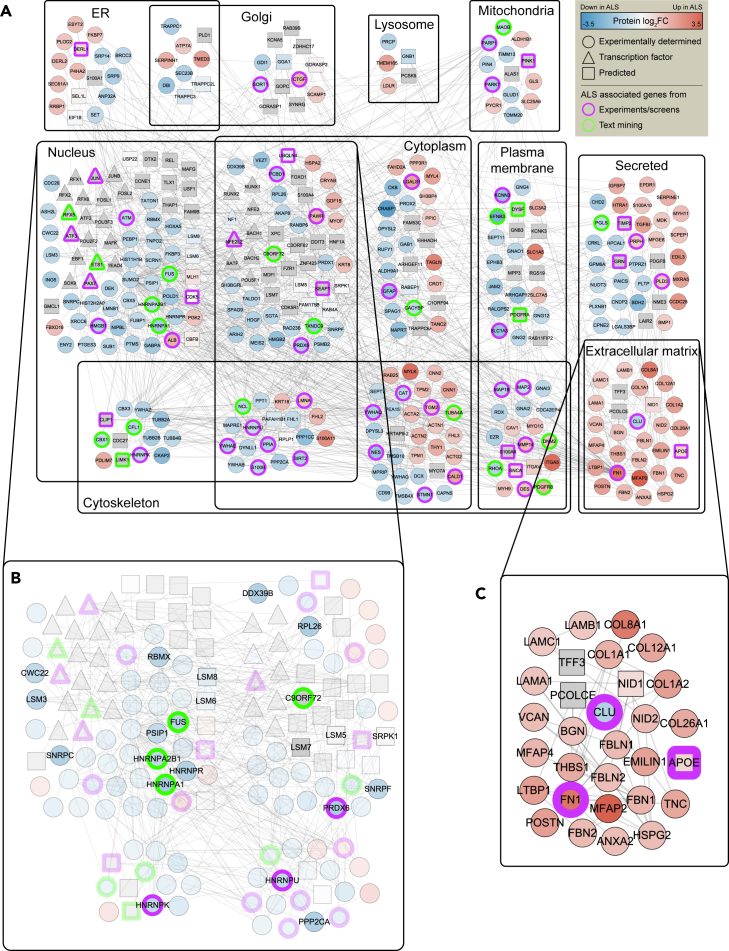
Data integration (A) Integrative analysis reveals a network of 374 proteins organized by subcellular location, of which 264 are experimentally determined from proteomics (circles), 27 are predicted transcription factors, and 83 are other proteins that were closely connected by physical interactions. Borders indicate ALS-associated proteins from experiments or screens (purple) and text mining (green). (B) A zoomed-in view of the nucleus compartment displaying genes with RNA metabolism functions. (C) A zoomed-in view of the extracellular matrix compartment.

Omics Integrator was used to search for connections among 376 predicted TFs and DEPs. After optimization and filtering for robustness, the network retained 291 of these proteins and added 83 proteins that were closely connected by physical interactions. The resulting 374-node network is shown in Figure 4A, with nodes organized by cellular compartment.

To evaluate the performance of our algorithm, we assessed the network for enrichment of genes previously associated with ALS (see Table S13 for ALS gene composition). We found strong enrichment for ALS-associated proteins (Figure 4A bolded borders; p value = 4.0 × 10^−13^). We also found that the 83 proteins added by Omics Integrator were also enriched for ALS-associated genes (p value = 2.4 × 10^−3^), providing confidence that our method can predict disease-relevant proteins and pathways.

In order to understand the function of the identified network, we scored it using categories from Gene Ontology. Enrichment analysis revealed significant dysregulation of ECM, similar to the transcriptomic, proteomic, and epigenomic results. Furthermore, the network was enriched for proteins belonging to cytoskeletal organization and RNA metabolism pathways (Figures 4A and 4B), both previously implicated in ALS. For instance, the nuclear-cytoskeletal compartment contains cofilin (CFL1), a known interaction partner of C9ORF72 that modulates actin dynamics in motor neurons (Sivadasan et al., 2016). LIMK1, a kinase that phosphorylates CFL1 also appears in the network and is known to also phosphorylate MMP14 (found in the cytoskeletal-plasma membrane compartment in Figures 4A and 4C), an endopeptidase that degrades ECM components (Lagoutte et al., 2016). Proteins involved in microtubule organization (PPP2CA, MAP1B, tubulin) are also represented in the cytoskeletal component of the network. PPP2CA, a major phosphatase for microtubule-associated proteins and a known binding partner of C9ORF72, has been shown to activate MAP1B, which in turn tyrosinates tubulin (Coyne et al., 2014). Our network also features mitochondrial proteins that are involved in responses to oxidative stress. Mutations in PARK7 have been linked to ALS (Hanagasi et al., 2016), and its knockdown has been shown to increase disease severity in SOD1 mouse models (Lev et al., 2015). Furthermore, PINK1, a PARK7 mitochondrial cofactor, plays a role in axonal transport of mitochondria (Moller et al., 2017). Lysosomal dysfunction has also been implicated in ALS (Hardiman et al., 2017). Small GTPase RAB39B plays an important role in the initiation of autophagy via C9ORF72’s GDP-GTP exchange factor activity (Corbier and Sellier, 2017). UBQLN4, linked to ALS and found in the cytoplasmic component of the network, may assist in maturation of autophagosomes (Edens et al., 2017).

The network also revealed potentially pathological interactions between differential proteins and predicted transcriptional regulators. SUMOylation via SUMO2 is a post-translational modification process that can affect structure, localization, activity, and stability of substrates. Specifically, SUMOylation of POU5F1 (Oct4) and PAX7 enhances their stability and transactivity (Luan et al., 2013; Wei et al., 2007), whereas SUMOylation of JUN (AP1 family), ETS1, and RUNX2 reduces their stability and transactivity (Bossis et al., 2005; Ji et al., 2007). The SUMO2 protein was downregulated in ALS samples, and the activity of these transcriptional regulators following SUMOylation is concordant with their predicted activity in Figure S15. SUMOylation’s role in affecting the stability of hnRNPs and localization of actin components to the nucleus has previously been reported (Hofmann et al., 2009; Lee et al., 2012). Finally, a recent study showed that SUMOylation of stress granule proteins is required for disassembly, which is impaired by C9ORF72-associated dipeptide repeats (Marmor-Kollet et al., 2020). Our analysis suggests that SUMOylation may have substantial influence on transcriptional regulation in C9-ALS motor neurons.

### Analysis of integrated network in human postmortem C9 cervical spine

In order to assess the statistical and biological rigor of our data, we compared our *network data* to RNA-Seq data from an independent cohort of 12 C9 and 10 control subject postmortem cervical spines (Prudencio et al., 2020) and found a large overlap between these DEGs (3,168 at FDR < 0.1) and our integrated network, especially of the ECM subnetwork (8 overlapping genes). To explore the possibility that the network optimization biased this result, we also computed an empirical p value, as follows. Omics Integrator was run on 100 randomized inputs, generating 100 randomized networks (see STAR Methods section for details). We then computed significance (Fisher’s exact test) of overlap between the network nodes (genes/proteins) and DEGs in the postmortem cervical spine (Table S9) for each randomized network and plotted this in Figure S16A. Figure S16B shows a density plot for the number of overlapping genes from the 100 randomized networks. The mean of the distribution of p values is shifted far from our true p value. Only one randomized network reached a significance of enrichment greater than our true network (empirical p value <0.01), with a large number (42) not even reaching significance at an alpha of 0.05. In addition, using 1,000 random permutations of patient condition labels for the postmortem data, we assessed the statistical significance of those DEGs (Figure S16C). Of 1,000 permutations, only 1 had a number of DEGs >3,168, indicating an empirical p value <0.001. These 1,000 sets of DEGs were then overlapped with the ECM subnet (30 genes) in the integrated network, and distribution of the number of overlapping genes is shown in Figure S16D. Of 1,000 permutations, none of the DEG lists have an overlap ≥8, indicating an empirical p value <0.001. Taken together, dysregulation of the ECM subnetwork from the iPSC-derived motor neuron study is observed in both the iPSC-derived subnetwork and in the human postmortem data.

### A fly screen validates key pathways

**Figure 5:**
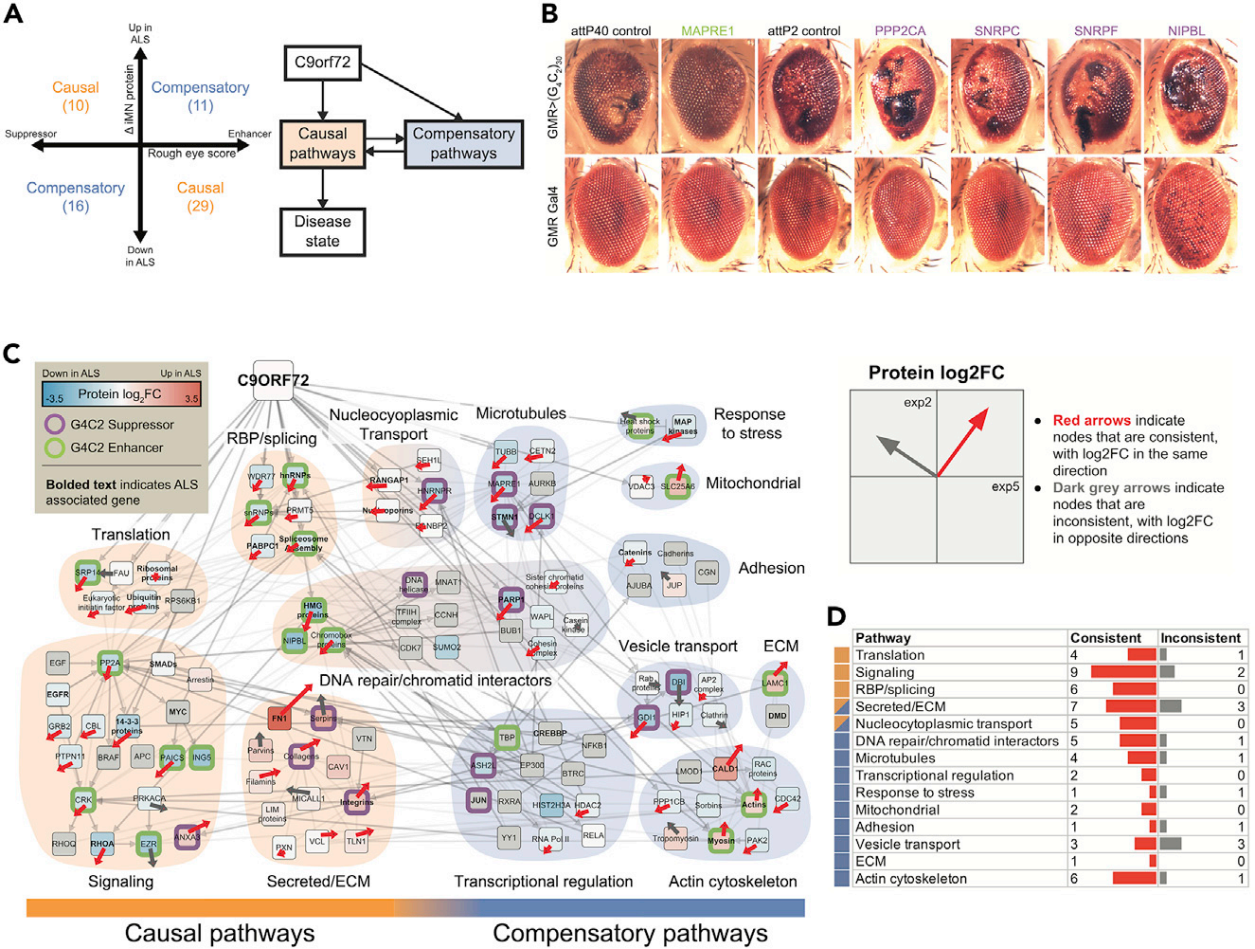
Validation in *Drosophila* (A) Left: Each gene that was tested in the fly model is sorted into causal or compensatory categories using its fly phenotype and change in protein values in iMNs. Right: A schematic showing the interplay between causal and compensatory pathways that eventually result in the disease. (B) The effect of genetic manipulations on external eye morphology and depigmentation in G4C2-expressing flies. (C) Causal and compensatory genes from A were connected via intermediate genes and the resulting network was organized by cellular process. Proteins from the same families were consolidated into a single node for readability. The borders indicate whether the gene is a G4C2 suppressor (purple) or enhancer (green). Bolded names indicate ALS-associated genes. The horizontal and vertical components of the arrows indicate protein fold changes (ALS/CTR) between the original and validation experiments, respectively. Red arrows indicate proteins whose fold changes were consistent between experiments, whereas dark gray arrows indicate proteins that were inconsistent. (D) Numbers of consistent and inconsistent nodes between the original and validation experiments within each pathway in the *Drosophila* network in (C).

In order to evaluate our “integrated -omics” list generated from control and C9-ALS iMNs *in vivo*, we conducted an RNAi-based screen in a *Drosophila* model of G4C2-mediated neurodegeneration (Xu et al., 2013). In this model, over-expression of 30 G4C2 repeats in the eye leads to age-dependent photoreceptor neurodegeneration, and genetic pathways identified as modifiers of fly eye degeneration have proven to be relevant to C9ORF72-associated neurodegeneration in mouse and human iPSC-derived neuron models (Xu et al., 2013; Zhang et al., 2016). Following identification of fly homologs of human genes identified in our -omics analyses (see STAR Methods), a total of 288 fly genes corresponding to 242 human genes were knocked down in the G4C2 fly model and their ability to modify (suppress or enhance) the rough eye phenotype was scored (Figures 5A and 5B). When available, multiple RNAi lines were tested. Of those, about 20% enhanced and 15% suppressed C9 toxicity (Table S14) with a score of at least ±1, respectively (Figure S17). The remainder showed little or no effect on eye degeneration and approximately 2% resulted in lethality. There was no particular relationship between the proteomic changes in iMNs and the phenotypic effect of knocking down the gene in the fly (Table S15). Although the precise mechanism by which these genetic manipulations affect neurodegenerative phenotypes in the fly eye are unknown at this time, the results from the fly screen confirm that a subset of genes/proteins, identified through our integrated -omics approach, can modify and/or contribute to C9ORF72 G4C2 repeat-mediated toxicity.

### Characterization of putatively causal and compensatory pathways

We leveraged the fly results to explore the potential causal roles of proteins that changed in the iMN data. Based on -omics data alone, where specific genes, proteins, and pathways are identified as up- or down-regulated, it is not possible to determine whether a difference in ALS versus control motor neurons is part of the toxic effects of the C9ORF72 expansion or whether it represents a compensatory process. However, we can begin to resolve this ambiguity using the results of the RNAi screens performed in the above fly model of the repeat expansion. For example, in the simplest case, if a protein is upregulated in C9-ALS motor neuron cultures and knockdown suppresses eye degeneration in the fly, the ALS-induced change(s) were likely deleterious. We refer to such C9-induced changes as “causal.” By contrast, if knockdown of the same protein resulted in enhanced eye degeneration, the ALS-induced change(s) are more likely to be part of a compensatory adaptation. In total, we found 39 causal and 27 compensatory genes (Figure 5A, and examples in Figure 5B).

We developed an integrative approach to discover the functional interactions among these genes and their underlying roles in ALS pathology. Specifically, we built networks connecting these proteins using directed interactions gathered from two public pathway databases—KEGG and Reactome (see STAR Methods)—and grouped the resulting proteins by functional categories (Figure 5A). This approach revealed several causal pathways (Figure 5C) that were previously known to be dysregulated by the mutated form of C9ORF72, such as RNA splicing and nuclear transport (Robberecht and Philips, 2013; Zhang et al., 2016). The altered proteins in these pathways include ALS-associated genes such as hnRNPA1, FUS (located in the Spliceosome Assembly node), and RanGAP1 (Table S13). Other pathways emerged as causal that have been less thoroughly examined in the context of C9ORF72. These pathways include signaling pathways such as EGF signaling, SMAD signaling (e.g., EZR and CRK) with a hub centered on phosphatase PP2A. The approaches used here also highlighted a causal set of ECM-related pathways and genes including integrins, collagens, and serpins. Within these networks based on the fly data, a number of pathways are likely to represent compensatory changes. For instance, the observed increases in the cytoskeletal proteins like actin, myosin, and tropomyosin; increases in heat shock proteins; and decreases in RAC proteins and other proteins relating to GTP/GDP exchange may be compensatory. Our approach also begins to reveal interactions between different processes. For example, the putatively causal toxic changes in the nucleocytoplasmic transport or oxidative stress are connected to potentially compensatory changes in DNA repair pathways. Finally, regulation of causal and compensatory processes can be elucidated using this approach (Figure 5C). As an example, ECM/secreted proteins fall into causal pathways, whereas cell adhesion protein changes are largely compensatory, as is dysregulation of Laminin C1, which is a component of the basal lamina and is secreted and incorporated into ECM matrices as an integral part of the structural scaffolding in tissues.

### Validation with a different motor neuron culture

We next asked whether the data integration could be validated using a replication cohort and tested our results using a different cellular model and with six patients with C9ORF72 and seven controls, none of which were in common with the original set of samples (Figure S4). To investigate the robustness of the original findings, we used a modified motor neuron differentiation protocol termed direct iPSC-derived Motor Neurons diMNs at 18 days of differentiation (Coyne et al., 2020a; Sances et al., 2016; Ho et al., 2020) (Figure S2) that comprised three main stages. In stage 1, neural induction and hindbrain specification of iPSCs is achieved by inhibition of dual SMAD and GSK3β pathways. During stage 2, specification of spinal motor neuron precursors is achieved by addition of sonic hedgehog agonists and retinoic acid. Maturation of these precursors into neurons with more complex processes and neurites occurs during stage 3 with addition of neurotrophins and Notch pathway antagonists. This protocol generated an equivalent overall neuronal composition consisting of ∼75%–80% of βIII-tubulin (TuJ1)-positive neurons in both CTR and C9-ALS diMNs cultures (Figures S2 and S3). The percentage of spinal motor neurons based on SMI32 (NEFH)- and NKX6.1-positive cells was also not statistically different between CTR and C9-ALS cultures—SMI32 (CTR = 59.7% and C9-ALS = 43.5%) and NKX6.1 (CTR = 39.3% and C9-ALS = 27.1%). However, a significantly different percentage of ISLET1-positive cells was observed in control (41.1%) versus C9-ALS (26.9%) cultures (p value = 0.0009) in day 18 diMNs (Figures S2 and S3).

RNA-Seq (PCA in Figure S8B) and proteomics were then performed on each of the samples. The differential signals for the original and validation experiments were correlated for RNA (Pearson = 0.25; p value = 8.9 × 10^−224^) and proteomics (Pearson = 0.24; p value = 1.7 × 10^−33^). RNA-Seq identified 91 differential transcripts (Table S9) that were enriched for actin cytoskeleton terms, similar to the enrichments from the original analysis. Of these 91 transcripts, 6 were in common with the original set of differential genes (p value = 0.0015). Proteomics also identified 250 differential proteins that were enriched for ECM, adhesion, and axon-related terms, although RNA binding and splicing terms were not found to be enriched. Of these 250 proteins, 68 were in common with the original set of differential genes (p value = 0.05).

Finally, we explored how well the validation data mirrored the original integrated network (Figure 4) by comparing the signed proteomics log fold change for each node in the network with proteomics from the validation data. Proteins were significantly more likely to be consistent between experiments, with 133 proteins changing in the same direction versus 40 proteins changing in opposite directions (p value = 1.5 × 10^−12^). More secreted/ECM proteins were consistent than not (11 versus 1), and more cytoskeletal proteins were consistent than not (40 versus 12) (Figures 5D and S18). The differential protein expression for nodes in the network were significantly correlated (Pearson = 0.49; p value = 5.5 × 10^−12^), indicating good concordance between the original and validation data.

## Discussion

iPSC models offer a way to map the initiation and execution of pathology in specific diseases of the central nervous system (CNS). This is clearly required given the lack of effective drugs for brain disorders despite years of investment from both industry and academia. Many groups have now been able to generate iPSCs from patients with neurological disease-causing mutations and have shown specific phenotypes in the dish (Consortium, 2017; Laperle et al., 2020), and we recently reviewed the many studies using iPSCs to model ALS (Sances et al., 2016). More recent studies show a stress-induced phenotype in C9 iPSC-derived motor neurons (Shi et al., 2018) and an overall cell death and reduced fiber outgrowth phenotype in a range of ALS cases not including C9ORF72 (Fujimori et al., 2018). In another report, increased activity in motor neurons from patients with ALS in the dish led to a drug trial with retigabine (Mcneish et al., 2015). Of interest, all these studies were focused on discovering *in vitro* phenotypes such as cell death or reduced fiber outgrowth, which may or may not be relevant to drug intervention in patients. One of the key difficulties in these studies has been an incomplete picture of the earliest and most significant changes that occur during pathogenesis.

Based on the premise that dysfunction of molecular pathways in specific cell populations in the brain leads to neurodegeneration, we have established a quantitative molecular phenotyping approach using a human iPSC technology platform to study molecular signatures of CNS cell types. We focused on iPSCs from patients with C9ORF72, given its prevalence as a genetic cause of ALS and its dominant phenotype (Brown and Al-Chalabi, 2017), to try to overcome variability that can arise between assay types and between different experiments. We have used genomics, transcriptomics, epigenomics, and high-content, quantitative proteomics to characterize motor neuron cultures from patients with C9ORF72 ALS, under strict quality control including the use of *parallel and identical* cultures for each assay, metadata standards, and analytical pipelines. The use of the same cultures was critical, given that cell type heterogeneity arising from iPSC differentiations and batch effects can be a confound in molecular analyses of long-term differentiations (Volpato et al., 2018). Given that the C9ORF72 mutation is a variable-sized intron expansion of G4C4 and that all unaffected people normally have variable numbers of repeats as well, the generation of isogenic lines that replace the expansion with a “normalized” repeat has been challenging. Furthermore, generation of isogenic lines are not feasible as most ALS cases are sporadic. Therefore, we used lines derived from different patients.

A computational pipeline was used to integrate the diverse molecular datasets and identify the most significant regulated pathways in patient cells. This Omics Integrator software (Tuncbag et al., 2016) uses network approaches to integrate diverse data types into coherent biological pathways that can avoid some of the pitfalls associated with analyzing single data types and uncover pathways that are not annotated in existing databases. This approach is validated by the strong statistical enrichment and the comprehensive number of hits it recovered that are consistent with the published literature for C9ORF72 ALS. The significant overlap between our integrated network, especially of the ECM subnetwork and data from C9ORF72 ALS postmortem cervical spine tissues, further suggests that this multi-omics iPSC-based motor neuron model may have relevance to changes that occur in the human spinal cord. At the same time, the approach revealed functional links among the disparate data, including identifying many transcriptional regulators.

A challenge in using multi-omic datasets is understanding how the direction of a change impacts disease pathogenesis. This is perhaps one of the greatest difficulties, e.g., understanding if the observed changes are conducive to the course of the disease or a cellular attempt at a homeostatic response to physiological insults. Using *Drosophila* genetics guided by the outcome of the integrated networks, it has been possible to not only validate the specific genes and proteins involved but also to discern probable effect and whether altered expression or activity would be predicted to promote disease pathogenesis or serve as a compensatory response. The results of these studies provide a unique data source and methods that can be utilized in the study of ALS and other neurodegenerative diseases.

Our analysis reveals a complex system of interweaving relationships among causal and compensatory pathways. In some cases, such as the ECM, causal and compensatory roles were found to exist even within the same pathways. Although the literature on the ECM's role in neuronal function and disease progression is limited, several studies have described neuroprotective properties of ECM (Suttkus et al., 2016) and a proteomic study of ALS subject cerebral spinal fluid revealed the ECM as an enriched biological process (Collins et al., 2015). Our analysis suggests that, although ECM components are broadly upregulated in ALS, individual components of the ECM may have very different downstream consequences. For example, knocking down some proteins like LAMC1 and DMD enhances toxicity in fly eyes, whereas knocking down other ECM components like serpins, collagens, and integrins suppresses toxicity. One mechanism through which extracellular signals within the ECM may be internalized is through integrin signaling. Integrin activation mediates molecular coupling of CAS and Crk, and the resulting complex has been shown to regulate the actin cytoskeleton (Chodniewicz and Klemke, 2004). Interestingly, integrins and CRK were both found to be pathogenic, whereas actin cytoskeletal components were compensatory, which suggests ECM pathogenicity may be transmitted via some non-cytoskeletal pathway.

It is also important to recognize that the classification of changes as “causal” or “compensatory” is far from definitive. Not all results from the fly necessarily translate to human cells and tissue. Furthermore, our simple binary classification does not capture complicated situations in which there may be non-linear effects of gene expression on phenotypes. However, these first attempts at relating many different aspects of cell functions are the starting blocks for further studies and enable a paradigm for generating a holistic view of cell functions in the face of a pathogenic repeat that underlies ALS.

Studies of human-derived samples must account for the variation that can result from differences in individuals as well as in cell state that may not be disease-related. In our study, we separately examined cells from two different cohorts that were differentiated using different protocols. Although the details of the DEGs and DEPs varied across the cohorts, our integrative approach was able to highlight many systems-level similarities. It is worth noting that large differences between cohorts is just as much an issue in studies of postmortem tissue as it is in studies of iPSC-derived material. For example, a recent proteomic analysis of Alzheimer’s postmortem brain samples found that, of 173 proteins initially detected as differentially expressed in the brain, only 58 showed consistent changes in a second cohort, and only 34 of these showed consistent changes in RNA levels (Bai et al., 2020). Focusing on network-level changes may help uncover commonalities that transcend differences among samples.

This integrative approach is well-suited for the task of hypothesis generation. For instance, our results suggest that DNA repair pathways are a compensatory response to either nucleocytoplasmic transport deficits or oxidative stress. In addition to providing insight into how these pathways interact, our analysis also identifies proteins that are attractive targets such as MAPRE1. Although we acknowledge there are some limitations of integrating data across human *in vitro* and fly *in vivo* models, this approach provides a much-needed basis for establishing causality and generating testable hypotheses.

An additional benefit in having transcriptomic and proteomic data together with WGS is the ability to integrate these datasets and identify whether a given DNA sequence change causes altered expression of the gene or altered levels of the protein. Using the dataset here, we have integrated WGS with RNA-Seq data to establish a pipeline and begin to evaluate eQTLs that may be meaningful to disease as a causal modifier versus altering gene expression as a consequence of ALS.

Future studies will expand this analysis across each of assays and extend to larger datasets from additional ALS subjects. The goal of this study was to establish a platform to evaluate multiple subjects and incorporate human variation by gathering and integrating a wide range of information using highly systematic approaches. Our approach to data integration was hierarchical, treating the proteomics, inferred gene regulation and gene expression as separate layers of abstraction. In this approach, we were able to infer key transcription factors, confirming that they are themselves transcriptionally regulated. Network integration revealed that these regulators are linked by protein-protein interactions to the measured proteomic changes and implicated SUMOylation as a potential aspect of the cellular response to the C9ORF27 expansion. Through genetic manipulation in the fly, we were also able to determine that several of these predicted regulators function to compensate for pathological changes induced by the C9ORF72 expansion.

### Limitations of the study

This study was underpowered with regard to numbers of patients and made no connection to the complex clinical course of the disease. Currently we are producing 1,000 iPSC lines from patients with all types of ALS (including *C9ORF72* mutation carriers) and performing similar analyses under the auspices of Answer ALS (https://www.answerals.org/) along with single-cell RNA-Seq on a subset of lines. In addition, the clinical history of each patient will be combined with the Omics Integrator and other computational approaches, including accounting for cell heterogeneity and gender as covariates in the analyses, to give more resolution on how molecular changes may impact the clinical course of the disease. However, the core techniques and integrated approach of the current report suggest a strategy to generate molecular disease signatures for ALS and provide a framework for this new “big data” approach to learning more about causes and treatments of diseases such as ALS.

## Consortia

The members of the NYGC ALS consortium are Hemali Phatnani, PhD, Justin Kwan, MD, Dhruv Sareen, PhD, James R. Broach, PhD, Zachary Simmons, MD, Ximena Arcila-Londono, MD, Edward B. Lee, MD, PhD, Vivianna M. Van Deerlin, MD, PhD, Neil A. Shneider, MD, PhD, Ernest Fraenkel, PhD, Lyle W. Ostrow, MD, PhD, Frank Baas, MD, PhD, Noah Zaitlen, PhD, James D. Berry, MD, MPH, Andrea Malaspina, MD, PhD, Pietro Fratta, MD, PhD, Gregory A. Cox, PhD, Leslie M. Thompson, PhD, Steve Finkbeiner, MD, PhD, Efthimios Dardiotis, MD, PhD, Timothy M. Miller, MD, PhD, Siddharthan Chandran, PhD, Suvankar Pal, MD, Eran Hornstein, MD, PhD, Daniel J. MacGowan, MD, Terry Heiman-Patterson, MD, Molly G. Hammell, PhD, Nikolaos. A. Patsopoulos, MD, PhD, Oleg Butovsky, PhD, Joshua Dubnau, PhD, Avindra Nath, MD, Robert Bowser, PhD, Matt Harms, MD, Mary Poss, DVM, PhD, Jennifer Phillips-Cremins, PhD, John Crary, MD, PhD, Nazem Atassi, MD, Dale J. Lange, MD, Darius J. Adams, MD, Leonidas Stefanis, MD, PhD, Marc Gotkine, MD, Robert H. Baloh, MD. PhD, Suma Babu, MBBS, MPH, Towfique Raj, PhD, Sabrina Paganoni, MD, PhD, Ophir Shalem, PhD, Colin Smith, MD, Bin Zhang, PhD, Brent Harris, MD, PhD, Iris Broce, PhD, Vivian Drory, MD, John Ravits, MD, Corey McMillan, PhD, Vilas Menon, PhD, Lani Wu, PhD, and Steven Altschuler, PhD.

## STAR★Methods

### Key resources table

**Table:** 

REAGENT or RESOURCE	SOURCE	IDENTIFIER
**Antibodies**		

mouse anti-SMI32	Covance	# SMI-32P; RRID:AB_2314912
mouse anti-TuJ1	Sigma	MAB1637; RRID:AB_2210524
rabbit anti-GFAP	Dako	Z0334; RRID:AB_10013382
mouse anti-Map2a/b	Sigma	M1406; RRID:AB_477171
rabbit anti-nestin	Millipore	ABD69; RRID:AB_2744681
Goat anti-human Islet-1	R&D	AF1837; RRID:AB_2126324
Rat anti-Nkx-6.1	DSHB	F55A10-s; RRID:AB_532378

**Chemicals, peptides, and recombinant proteins**		

Hoechst 33258	Sigma	33258
IMDM	Life Technologies	12440061
F12	Life Technologies	11765062
NEAA	Life Technologies	11140-50
B27	Life Technologies	17504044
N2	Life Technologies	17502048
Anti/Anti	Life Technologies	15240062
LDN193189	Cayman Chemical	19396
CHIR99021	Xcess bioscience	M60002
SB431542	Cayman Chemical	13031
All-trans RA	Stemgent	04-0021
SAG	Cayman Chemical	11914
Rock Inhibitor (Y-27632)	Stemcell Technologies	72308
db-cAMP	Millipore	28745
Compound E	Calbiochem	565790
DAPT	Cayman Chemical	13197
Ascorbic Acid	Sigma-Aldrich	A4403
BDNF (-80)	Peprotech	450-02
GDNF (-80)	Peprotech	450-10

**Critical commercial assays**		

QIAamp DNA Blood mini Kit	Qiagen	51104
Qiagen RNeasy mini kit	Qiagen	74104
Ribo-Zero Gold rRNA depletion and Truseq Stranded total RNA kit	Illumina	20020598
Biognosys iRT mixture	Biognosys	Ki-3002-2
Expedeon FASP protocol	Abcam	ab270519
BCA assay	Pierce	23227
Nextera XT DNA Library Preparation Kit	Illumina	FC-131-1096

**Deposited data**		

ATAC-seq	This paper	http://lincsportal.ccs.miami.edu/dcic-portal/
RNA-Seq	This paper	http://lincsportal.ccs.miami.edu/dcic-portal/
Proteomics	This paper	https://chorusproject.org/pages/dashboard.html#/search/Neurolinc/projects
Whole-Genome Sequencing	This paper	http://data.answerals.org/search#root-neurolincs

**Experimental models: Cell lines**		

Control human iPSC 25iCTR	The Cedars-Sinai Biomanufacturing Center (iPSC Core)	CS25iCTR-18nxx
Control human iPSC 83iCTR	The Cedars-Sinai Biomanufacturing Center (iPSC Core)	CS83iCTR-33nxx
Control human iPSC 00iCTR	The Cedars-Sinai Biomanufacturing Center (iPSC Core)	CS00iCTR-nxx
ALS human iPSC 29iALS	The Cedars-Sinai Biomanufacturing Center (iPSC Core)	CS29iALS-C9nxx
ALS human iPSC 52iALS	The Cedars-Sinai Biomanufacturing Center (iPSC Core)	CS52iALS-C9nxx
ALS human iPSC 30iALS	The Cedars-Sinai Biomanufacturing Center (iPSC Core)	CS30iALS-C9nxx
ALS human iPSC 28iALS	The Cedars-Sinai Biomanufacturing Center (iPSC Core)	CS28iALS-C9nxx
Control human iPSC 002iCTR	The Cedars-Sinai Biomanufacturing Center (iPSC Core)	CS0002iCTR-nxx
Control human iPSC 0179iCTR	The Cedars-Sinai Biomanufacturing Center (iPSC Core)	CS0179iCTR-nxx
Control human iPSC 0201iCTR	The Cedars-Sinai Biomanufacturing Center (iPSC Core)	CS0201iCTR-nxx
Control human iPSC 0206iCTR	The Cedars-Sinai Biomanufacturing Center (iPSC Core)	CS0206iCTR-nxx
Control human iPSC 1ATZiCTR	The Cedars-Sinai Biomanufacturing Center (iPSC Core)	CS1ATZiCTR-nxx
Control human iPSC 1WP3iCTR	The Cedars-Sinai Biomanufacturing Center (iPSC Core)	CS1WP3iCTR-nxx
Control human iPSC 9XH7iCTR	The Cedars-Sinai Biomanufacturing Center (iPSC Core)	CS9XH7iCTR-nxx
ALS human iPSC 0BUUiALS	The Cedars-Sinai Biomanufacturing Center (iPSC Core)	CS0BUUiALS-nxx
ALS human iPSC 2YNLiALS	The Cedars-Sinai Biomanufacturing Center (iPSC Core)	CS2YNLiALS-nxx
ALS human iPSC 6UC9iALS	The Cedars-Sinai Biomanufacturing Center (iPSC Core)	CS6UC9iALS-nxx
ALS human iPSC 6ZLDiALS	The Cedars-Sinai Biomanufacturing Center (iPSC Core)	CS6ZLDiALS-nxx
ALS human iPSC 7VCZiALS	The Cedars-Sinai Biomanufacturing Center (iPSC Core)	CS7VCZiALS-nxx
ALS human iPSC 9YHNiALS	The Cedars-Sinai Biomanufacturing Center (iPSC Core)	CS9YHNiALS-nxx

**Experimental models: Organisms/strains**		

*D. melanogaster*: knocking-down or overexpressing these genes downstream of UAS sites for GAL4-specific modulation	Bloomington Drosophila Stock Center	

**Software and algorithms**		

Burrows-Wheeler Aligner BWA-MEMv0.7.8	Li and Durbin, 2009	http://bio-bwa.sourceforge.net/
Picard tools (v1.83)	http://broadinstitute.github.io/picard/	http://picard.sourceforge.net
Genome Analysis Toolkit (GATK v3.4.0)	DePristo et al., 2010; McKenna et al., 2010	https://gatk.broadinstitute.org/hc/en-us
HTSeq	Anders et al. (2015)	https://htseq.readthedocs.io/en/master/overview.html
DESeq2	Love et al. (2014)	https://bioconductor.org/packages/release/bioc/html/DESeq2.html
Ingenuity pathway analysis	https://www.qiagenbioinformatics.com/products/ingenuitypathway-analysis	https://digitalinsights.qiagen.com/products-overview/discovery-insights-portfolio/analysis-and-visualization/qiagen-ipa/
Gorilla	Eden et al. (2009)	http://cbl-gorilla.cs.technion.ac.il/
Cytoscape	Otasek et al., 2019	https://cytoscape.org/
edgeR	Robinson et al., 2010	https://bioconductor.org/packages/release/bioc/html/edgeR.html
OpenSWATH	http://openswath.org/en/latest/index.html	http://openswath.org/en/latest/
HOMER	Heinz et al. (2010)	http://homer.ucsd.edu/homer/motif/
MACS2	Zhang et al. (2008)	
Image-Pro Insight v9	https://www.mediacy.com/support/imageproinsight	https://www.mediacy.com/support/imageproinsight
ProteoWizard v.3.0.6002	Kessner et al., 2008	https://sourceforge.net/p/proteowizard/mailman/proteowizard-support/?viewmonth=201407
Trans Proteome Pipeline v.4.8	Keller et al., 2005	http://tools.proteomecenter.org/software.php
OmicsIntegrator2 package (v2.3.1)	Tuncbag et al. (2016)	https://github.com/fraenkel-lab/OmicsIntegrator2/

### Resource availability

#### Lead contact

Further information and requests for resources and reagents should be directed to and will be fulfilled by the lead contact, Leslie M. Thompson (lmthomps@uci.edu).

#### Materials availability

All of the iPSC lines described in this study are available from the iPSC Core at the Cedars-Sinai Biomanufacturing Center iPSC repository.

### Experimental model and subject details

#### Generation and characterization of iPSC lines

The initial 3 control lines (termed 25iCTR, 83iCTR, 00iCTR) and 4 iPSC lines (termed 29iALS, 52iALS, 30iALS, 28iALS) were generated using episomal plasmids and characterized as previously described (Sareen et al., 2013). Fibroblasts from C9ORF72 ALS patients (28iALS-n2, 29iALS-n1, 30-iALS-n1 and 52iALS-n6) were derived at Washington University of St. Louis. Healthy control fibroblasts (00iCTR: GM05400; 83iCTR: GM02183) were obtained from the Coriell Institute for Medical Research. The Coriell Cell Repository maintains the consent and privacy of the donor of fibroblast samples. All the cell lines and protocols in the present study were carried out in accordance with the guidelines approved by institutional review boards at the Cedars-Sinai Medical Center and Washington University at St. Louis. Studies were performed under the auspices of the Cedars-Sinai Medical Center Institutional Review Board (IRB) approved protocol Pro00028662 and Pro00028515. The reprogramming and characterization of iPSC lines and differentiation protocols in the present study were carried out in accordance with the guidelines approved by Stem Cell Research Oversight (SCRO) committee and IRB, under the auspices of IRB-SCRO Protocols Pro00032834 (iPSC Core Repository and Stem Cell Program), Pro00024839 (Using iPS cells to develop novel tools for the treatment of SMA) and Pro00027006 (Cell and Tissue Analysis for Neurologic Diseases; Robert Baloh). Appropriate informed consents were obtained from all the donors. Additional iPSC lines for the replicate cohort were generated from 7 healthy controls (CS-002, LBC360179, W15-C201, W15-C206, NEUMN061ATZ, NEUVW301WP3, NEUPW469XH7) and 6 ALS patients (NEUEM720BUU, NEUVX902YNL, NEUUL256UC9, NEUPK546ZLD, NEUFV237VCZ, NEUDT709YHN) and were reprogrammed from PBMCs. All lines were reprogrammed by nucleofecting parent cells with nonintegrating oriP/EBNA1 plasmids, which allowed for episomal expression of reprogramming factors similar to previously published protocols (Laperle et al., 2020; Barrett et al., 2014). All of the iPSC lines described in this study are available from the iPSC Core at the Cedars-Sinai Biomanufacturing Center iPSC repository. To protect donor privacy and confidentiality, all samples were coded and de-identified in this study. To assess purity and confirm donor identity of parental tissue (fibroblasts and blood), reprogrammed iPSCs and differentiated motor neurons, extensive quality control was implemented, including DNA fingerprinting and short tandem repeat (STR) analysis performed by IDEXX BioResearch. G-band karyotyping was performed to ensure that iPSCs maintained normal karyotypes. Each of the iPSC lines used in this study had unique genetic profiles and the profiles of the samples and their source tissues were identical. Additionally, the test confirmed the samples to be of human origin and detected no mammalian interspecies contamination. The Cedars-Sinai iPSC Core Facility created a working cell bank of iPSC-derived motor neuron precursor spheres (iMPS) and terminal diMNs for C9-ALS and control subjects.

#### Differentiation of iPSCs into motor neurons

*Initial Cohort:* Control and ALS iPSCs were differentiated into motor neurons (iMNs) based on a combination of previous models established for rapid neural differentiation (Figures 1A and S1) (Sances et al., 2016). Briefly, iPSCs were grown to near confluence devoid of spontaneous differentiation under normal maintenance conditions prior to the start of differentiation. Neuroectoderm specification of iPSCs was induced by removal of mTeSR1 media and addition of defined neural differentiation media (NDM) +LS composed of IMDM supplemented with B27 + vitamin A (2%), N2 (1%), Non-Essential Amino Acids (NEAA, 1%) and penicillin-streptomycin-amphotericin (PSA, 1%) along with LDN193189 and SB431542 [LS] - as a combination of small molecule inhibitors of SMAD pathway, BMP type 1 receptors (ALK2/3) TGF-beta superfamily type 1 activin receptor-like kinase (ALK) receptors (ALK4/5/7)] (Figure S1A). Colonies were dissociated into single cells with Accutase and uniform aggregates were formed in sterilized V-bottom 384-well PCR plates with 20,000 cells/well. Uniform neural aggregates were formed by seeding in NDM + LS in presence of Matrigel and centrifuging for 5 minutes at 200g. The aggregates were maintained in this media for 5 days. The culture medium was replenished every 2 days. On day 5, the aggregates were gently isolated from the plates using Accutase, and the uniform sized neural aggregates were then plated on laminin-coated 6-well plates. After 7 days (day 12), media were changed to a motor neuron specification medium (MNSM) generating caudo-ventralized MN precursors by addition of all-trans retinoic acid (ATRA) (0.25 μM) and the sonic hedgehog agonist, purmorphamine (PMN) (1 μM), brain-derived neurotrophic factor (BDNF) (20 ng/ml), glial cell line-derived neurotrophic factor (GDNF) (20 ng/ml), ascorbic acid (AA) 200 ng/ml) and dibutyryl cyclic adenosine monophosphate (db-cAMP) (1 μM). Over the next 4 to 8 days, neural rosettes formed and were lifted at day 16 to 20 and subsequently cultured in suspension low-attachment flasks for a further 8 days. Selected rosettes were switched to a motor neuron precursor expansion media (MNPEM) containing ATRA (0.1 μM), PMN (1 μM), and the mitogens epidermal growth factor (EGF) (100 ng/ml) and fibroblast growth factor (FGF2) (100 ng/ml). After an initial 8 days in the expansion medium, the induced motor neuron precursor spheres (iMPS) were further expanded by weekly chopping for 5 weeks (passages) and cryopreserved prior to initiation of terminal differentiation stage. These iMPS were cryopreserved into aliquots for later generation of iMPS-derived motor neurons (iMNs) for -omic analysis. In order to induce terminal motor neuron differentiation, the iMPS were fully dissociated with Accutase and seeded on laminin-coated 6-well plates, and matured in Stage 1 motor neuron maturation medium (MNMM Stage 1) consisting of NDM supplemented with ATRA (0.1 μM), PMN (1 μM), db-cAMP (1 μM), AA (200 ng/ml), Notch signaling γ-Secretase Inhibitor, DAPT (2.5 μM), BDNF (10 ng/ml) and GDNF (10 ng/ml) for 7 days (Figure S1B). Then cultures were switched to maturation medium Stage 2 (MNMM Stage 2) containing Neurobasal, 1% NEAA, 1% N2, 0.5% GlutaMax, db-cAMP (1 μM), AA (200 ng/ml), BDNF (10 ng/ml) and GDNF (10 ng/ml) for another 14 days. Mature iMN cultures were harvested and screened at 21-days post-plating. These conditions allowed for motor neuron differentiation under serum-free conditions. All differentiating cultures were maintained in humidified incubators at 37°C (5% CO2 in air).

*Replication Cohort:* A second differentiation method called “the direct induced motor neuron (diMN) protocol” which comprises three stages (Figure S2A) was used for the replication cohort studies. At the outset of Stage 1, plates from each iPSC line were washed with 1mL Dulbecco’s Phosphate-Buffered Saline (DPBS) (Corning 21-031-CV)/well and then incubated in 1mL Accutase (EMD Millipore SCR005)/well for 5 minutes at 37°C. After incubation, 1mL DPBS/well was added, cells were quickly collected into multiple 15mL conical tubes and centrifuged at 161g for 2 minutes. Each pellet was re-suspended in mTeSR and cell viability and concentration were determined by automated cell counting (Nexcelom Auto 2000), and multiple matrigel-coated 6-well plates were seeded at a density of 5e5 cells/well in 2 mL mTeSR media/well. Twenty-four hours following plate-down, mTeSR media was exchanged for Stage 1 media (Table S1 for media composition). Stage 1 media was exchanged daily until Day 6. On day 6, stage 2 of the differentiation process began when, for each cell line, all wells were washed with 1mL DPBS/well and incubated in 1mL Accutase per well for 5 minutes at 37°C. After incubation, 1mL DPBS/well was added, cells were quickly collected into multiple 15mL conical tubes and centrifuged at 161 g for 2 minutes. Cells were re-suspended in Stage 2 Plate Down Media (ST2PD, Table S2 for composition), viability and cell counts determined and multiple Matrigel-coated 6-well plates were seeded at a density of 7.5e5 cells/well in 2mL ST2PD/well. Twenty-four hours following plate down, St2PD was exchanged for Stage 2 media (Table S3 for composition). Stage 2 media were exchanged every other day until day 12. On day 12 began Stage 3 of differentiation. For each cell line, Stage 2 media was completely aspirated from all wells and replaced with 2 mL Stage 3 media/well. Stage 3 media (Table S4 for composition) was exchanged every other day until Day 18 of differentiation. During feedings, approximately 75% of old media was aspirated and 2 mL Stage 3/well was added dropwise in a circular manner in order to minimize disruption of the cell monolayer. On Day 18 of differentiation, cell lines were collected and pelleted. Prior to collection and pelleting, one 6-well plate or a 96-well plate seeded in parallel and carried through the entire protocol was selected from each line for immunocytochemistry-based quantification of select motor neuron and pan neuron markers including SMI32 (NEFH), Islet1, Nkx6.1, and TuJ1 (TUBB3) (Molecular Devices ImageExpress Micro) (Figures S2B and S3). Multiple regions (9–16) of interest were captured per well for four wells at a magnification of 10×. After imaging, the plates were collected with their respective lines.

Multiple wells/cell line were set aside for short tandem repeat (STR) analysis. For all remaining adherent wells, Stage 3 media was aspirated and replaced with 1mL DPBS/well. Adherent cell monolayers were manually scraped with a cell scraper (Falcon #353085) and collected using a serological pipette into 15mL conical tubes. Typically, two 6-well plates were collected per 15mL conical, and up to eight 6-well plates were collected per line. The 15 mL conical tubes were centrifuged for 2 minutes at 161 g. The supernatant was then aspirated and discarded, and the pellets were re-suspended in 1mL DPBS by gentle trituration using a P-1000 pipette. Once resuspended, all pellets were combined in a final volume of approximately 10 mL DPBS and centrifuged for 2 minutes at 161g . Again, the supernatant was aspirated and discarded. The pellet was then resuspended in 6mL DPBS using a 5 mL serological pipette and aliquoted to six 1.7 mL Eppendorf tubes (1 mL/Eppendorf tube). The Eppendorf tubes were centrifuged for 4 minutes at 161 g, and the supernatants were aspirated and discarded. Four of the Eppendorf tubes were snap frozen in an ethanol/dry ice slurry and stored at −80°C until shipment to -omics centers for analysis. The remaining two pellets were re-suspended in 1mL each of CryoStor CS10 (Biolife Solutions #210102) using a P-1000 pipette (typically, 2-4 triturations were sufficient to resuspend the pellets) and each pellet was transferred to an individual cryovial (Thermo Scientific #5000-1020). CryoStor vials were then stored in a Mr. Frosty (Nalgene #5100-0001) at −80°C for 24 hours, at which time they were transferred to sample boxes and stored at −80°C until shipment to -omics center for processing.

#### Animal models

*Drosophila melanogaster Screen*: An initial set of 249 genes were selected from two sources: 1) proteins from the integrated network and 2) miscellaneous genes of interest that were of interest to various group members. Out of the 249 genes, a majority (141) were selected from the integrated network. Drosophila orthologs of human DEGs were identified using DIOPT (Hu et al., 2011), and transgenic fly lines knocking-down or overexpressing these genes downstream of UAS sites for GAL4-specific modulation were obtained from the Bloomington Drosophila Stock Center. 300 fly genes were identified corresponding to these 249 human genes (or 284 including paralogues) and 334 total fly experiments were conducted (several fly genes were tested with different knockouts). Of these 334 total experiments, 7 exhibited lethal phenotype, 7 were not true modifiers as determined by GMR GAL4 score, and 1 was both. Filtering these out, there were 321 remaining fly experiments representing 288 fly genes and 242 human genes. These modifiers were crossed to flies overexpressing the hexanucleotide repeat expansion (HRE) in the eye [GMR Gal4; UAS-(G4C2)30/CyO]. Progeny co-expressing both the HRE and putative modifier were collected within 24 hours of eclosion and aged at 25°C and compared to control flies of the same genetic background. A relative modification index, ranging from −4 to +4, was used to assess eye degeneration where −4 represented complete rescue and +4 represented no eye (Zhang et al., 2015). A score of 0 represents no effect of the tested modifier. Ommatidial structure, interommatidial bristles, necrosis, loss of pigmentation, and overall morphology of the eye were assessed during scoring. Only female flies were scored due to male flies displaying a higher degree of variability. All experimental modifiers were tested with 3 biological replicates with their eye degeneration scores averaged. If a fly cross failed to eclose, the subsequent score was marked ‘lethal’. Selected strong enhancers and suppressors were retested with GMR Gal4; UAS-(G4C2)30/CyO as well as GMR Gal4 alone, at both 25°C and 29°C. At 15 days, representative female eyes were imaged using a Nikon SMZ1500 stereomicroscope and Lumenera INFINITY3-6UR 3.0 Megapixel camera and analyzed with Image-Pro Insight v9.

In some cases, a human candidate gene had multiple fly orthologs. For each human gene, a “weighted eye score” was calculated by taking the average of all corresponding fly orthologs, weighted by the ortholog scores as determined by the DRSC Integrative Ortholog Prediction Tool (https://www.flyrnai.org/cgi-bin/DRSC_orthologs.pl). Note that only moderate and high ranking orthologs were considered.

### Method details

#### Whole-genome sequencing and analysis

DNA was extracted from iPSC lines using the QIAamp DNA Blood mini Kit (Qiagen; 51104) as per the manufacturer's instructions. A minimum of 1 μg of unamplified, high molecular weight, RNase treated DNA with absorbance values of OD260/280 1.7- 2.0 and OD260/230 >2.0, was sent to The New York Genome Center for sequencing on the Illumina X10. Sequence data was processed on NYGC automated pipeline. Paired-end 150 bp reads were aligned to the GRCh37 human reference using the Burrows-Wheeler Aligner (BWA-MEMv0.7.8) and processed using the GATK best-practices workflow that includes marking of duplicate reads by the use of Picard tools (v1.83, http://picard.sourceforge.net), local realignment around indels, and base quality score recalibration (BQSR) via Genome Analysis Toolkit (GATK v3.4.0) (McKenna, Hanna et al. 2010; DePristo, Banks et al. 2011) (New York Genome Center).

The variant calls from NYGC were assessed by examining the actual reads for alignment issues and spot-checking the BAM files for specific variants in IGV and assessed they were of good quality. The VCFs were converted into GVCFs and performed custom annotation and intersected a subset of the omics data (RNA-Seq, ATAC Cluster) with the WGS data.

The annotation pipeline was customized to incorporate elements from ANNOVAR (Wang et al., 2010) and KGGseq (Li et al., 2012) and used to generate a report, including genotypes, for each sample. These reports are available upon request. The following annotation was used: For genes and exonic variants that have clinical significance, we incorporated the Clinical Genomic Database (CGD) (Solomon et al., 2013), the Online Mendelian Inheritance in Man (OMIM) (Amberger et al., 2015), ClinVar (Landrum et al., 2016) and genes listed in the American College of Medical Genetics and Genomics (ACMG) (Green et al., 2013) database as well. Intervar, which is based upon the ACMG and AMP standards and guidelines for interpretation of variants, was also incorporated. This tool uses 18 criteria to assess the clinical significance of variants and classify them based on a five- tiered system (Farrer et al., 1997). To flag ALS genes, we incorporated ALS gene lists and variants from ALSoD (Abel et al., 2013) (http://alsod.iop.kcl.ac.uk/), a highly curated list from Dr. John Landers and ALS associations from the DisGeNet database (Pinero et al., 2017). We also incorporated functional prediction by using *in silico* prediction from nine programs, including the databases, such as SIFT (Sim et al., 2012), PolyPhen2 (Chun and Fay, 2009), and Mutation Taster (Schwarz et al., 2010) and as in Li et al. (2013) for each variant. As well, additional databases were included that assess the variant tolerance of each gene using the RVIS (Petrovski et al., 2013), the Gene Damage Index (GDI) (Itan et al., 2015) and LoFTool (Fadista et al., 2017). Gene expression: For variants in genes that are highly expressed in the brain, we provided these data from the Human Protein Atlas (Uhlen et al., 2015) (http://www.proteinatlas.org) and expression data for the cortex and spinal cord from the GTEx portal (2013, 2015) (https://gtexportal.org/home/). Frequency information came from three databases on all known variants from ExAC (Lek et al., 2016), the NHLBI Exome Sequencing Project (ESP) (Tennessen et al., 2012), and the 1000 Genomes Project (Auton et al., 2015).

A separate annotation pipeline was developed for variants that are in intergenic and regulatory regions. We report the variant in relation to the closest gene, and are either intronic, upstream, downstream (up to 4 KBs from the start and stop of a gene) or in 5′ or 3′ UTRs. The annotation used came from RegulomeDB, which annotates variants with known or predicted regulatory elements such as transcription factor binding sites (TFBS), eQTLs, validated functional SNPs and DNase sensitivity (Boyle et al., 2012). The source data comes from ENCODE (2004, 2012) and GEO (Barrett et al., 2009). We also included other regulatory databases, such as Target Scan, which is an algorithm that uses 14 features to predict and identify microRNA target sites within mRNAs (Agarwal et al., 2015) and miRBase (Griffiths-Jones, 2004; Griffiths-Jones et al., 2006, 2008).

#### Immunocytochemistry

Human iPSC-derived motor neuron cultures were plated on optical-bottom 96-well plates (Thermo, # 165305) and subsequently fixed in 4% paraformaldehyde for 15 minutes. Cells were blocked in 5% normal donkey serum with 0.1% Triton X-100 in PBS and incubated with primary antibodies for 1 h at room temperature or overnight at 4°C. Cells were then rinsed and incubated in species-specific AF488, AF594, or AF647-conjugated secondary antibodies followed by Hoechst 33258 (0.5 μg/mL; Sigma) to counterstain nuclei. Cells were imaged using Molecular Devices ImageExpress Micro high-content imaging system or using Leica microscopes (Fuller et al., 2015) (Figure 1B). Primary antibodies used were as follows: mouse anti-SMI32 (Covance, 1:1,000); mouse anti-TuJ1 (β3-tubulin) (Sigma; 1:1,000-1:2,000); rabbit anti-GFAP (Dako; 1:1000); mouse anti-Map2a/b (Sigma; 1:1000); rabbit anti-nestin (Millipore; 1:2000), Islet-1 Antibody (R&D AF1837; 1:250) and Nkx-6.1 (DSHB F55A10-s; 1:100).

#### RNA-Seq

Total RNA was isolated from each sample using the Qiagen RNeasy mini kit. RNA samples for each subject (control or disease) were entered into an electronic tracking system and processed at the University of California, Irvine GHTF. RNA QC was conducted using an Agilent Bioanalyzer and Nanodrop. The primary QC metric for RNA quality is based on RIN values (RNA Integrity Number) ranging from 0-10, 10 being the highest quality RNA. Additionally, QC data was collected on total RNA concentration and 260/280 and 260/230 ratios to evaluate any potential contamination. Only samples with RIN >8 were used for library prep and sequencing. Library prep processing was initiated with total RNA of 1μg using a Ribo-Zero Gold rRNA depletion and Truseq Stranded total RNA kit. Additionally, ERCC exFold spiked-in controls were used for further QC and downstream data analysis. Briefly, RNA was chemically fragmented and subjected to reverse transcription, end repair, phosphorylation, A-tailing, ligation of barcoded sequencing adapters, and enrichment of adapter-ligated cDNAs. RNA-Seq libraries were titrated by qPCR (Kapa), normalized according to size (Agilent Bioanalyzer 2100 High Sensitivity chip). Each cDNA library was then subjected to Illumina (HiSeq 2500) paired end (PE), 100 cycle sequencing to obtain approximately 50-65M PE reads. After sequencing, raw fastq files were subject to QC measures and reads with quality scores (>Q20) collected and analyzed using the pipeline described at http://neurolincs.org/pipelines/. Briefly, reads were mapped to the GRCh37 reference genome, QCed, and gene expression and differential expression were quantified using tools HTSeq (Anders et al., 2015) and DESeq2 (Love et al., 2014). Normalized and transformed count data were then used for exploratory analysis and DE genes (FDR <0.1) were used for pathway, network, and gene ontology analysis. These primary data were subject to additional statistical and network-based data analyses using commercial and open-source pathway and network analysis tools, including IPA, GOrilla, Cytoscape, and other tools to identify transcriptional regulators, predict epigenomic changes, and determine potential downstream pathway and cellular functional effects. Significant DEGs (FDR<0.1) were then analyzed against genes that were found to contain exonic enriched genetic variants from the WGS. The gene expression (voom normalized and transformed values) and genotype variant pairs were analyzed by fitting a linear regression model. Adjusted R^2^ and Benjamini-Hochberg adjusted p-values were calculated, significant genes were reported at FDR < 0.1. The replication cohort was carried out using the same methods.

#### Proteomics

Frozen cell pellets were lysed using a combination of lysis buffer containing SDS and sonication. BCA assay was used to determine protein concentration and 125μg of each sample was used in downstream sample processing. Samples were processed following Expedeon FASP protocol (Wisniewski et al., 2009). Samples were digested in Trypsin/LysC (Promega) at a ratio of 40:1 to protein concentration at 37°C for 12 hrs. Samples were desalted using MCX micro-elution column (Waters) and samples were dried in speedvac and stored in −20°C until resuspension with Biognosys iRT mixture for acquisition on the SCIEX 6600 over a 45-minute gradient. Samples were acquired in data-dependent acquisition (DDA) mode for library building and in data-independent acquisition (DIA) mode over 100 variable windows similar to acquisition protocols in Kirk et al. (2015) and Holewinski et al. (2016). DDA files were run through Trans Proteome Pipeline (TPP) using a human canonical FASTA file (Uniprot). A consensus peptide library with decoys was generated. DDA library build principals as described in Parker et al. (2016) were utilized to generate a cell-specific library, which allowed for more accuracy in matching DIA data to the DDA library during OpenSWATH, as indicated by higher d-scores in PyProphet. DIA files were mapped onto this library using OpenSWATH and transition level data was compiled with a 1% FDR cutoff. Downstream summing of transition level data to peptide and protein level data was performed by MAP DIA (Teo et al., 2015). Log2FC data was calculated by MAP DIA and filtered using a 1% FDR, 95% confidence interval and 0.6 abs(log2FC) cutoff to obtain a final list of differentially expressed proteins. For protein quantification, transitions and peptides common to more than one protein were excluded. These data have been further analyzed using commercial and open-source pathway and network analysis tools, including Ingenuity pathway analysis and GOrilla to identify upstream regulators and determine affected cellular pathways.

*Replication Cohort:* The sample processing methodology for proteomic analyses was altered to enable high-throughput automation using the Biomek i7 Liquid Handling Automated Workstation. This concomitantly reduces manual processing and technical variations and thus improves long-term longitudinal aspirations inherent to this and ongoing projects. Cell pellets were lyophilized at −55°C and were solubilized in 6M Urea, 1 mM DTT in 1M NH4HCO3, pH 8 and sonicated at 70% amp, 10 sec on and 10 sec off (800R1 QSONICA) at 4°C. Sample volume was diluted by 2/3 using 100mM Tris, 4mM CaCl, pH 8. 200 μg protein, as determined by BCA assay (Pierce BCA Protein Assay Kit), was then transferred to a 96-well reaction plate for robotic digestion on the Biomek i7 automated workstation (Beckman Coulter). Reduction and alkylation was performed using TCEP and IAA, respectively and 2 μg of β-Galactosidase was added to each sample as a digestion control. Protein sample extracts were digested with 5 μg Trypsin/LysC mix (Promega) for 4 hours at 37°C. Final sample was acidified with 10% Formic Acid (FA) and transferred onto a conditioned 96-well HLB 5 mg column (Waters) for desalting. Peptides were eluted from the HLB with 50% MeCN, 0.1% FA and stored at −80°C after being dried to completion on a speedvac system. Mass spectral DIA data was generated on the Triple TOF 5600 (SCIEX) and the peptide ion library data was generated on the Triple TOF 6600 (SCIEX). Data analysis was performed as described above.

#### ATAC-seq

The assay for transposase-accessible chromatin using sequencing (ATAC-Seq) was used to assess chromatin accessibility and identify functional regulatory sites involved in driving transcriptional changes associated with C9ORF72. ATAC-seq detects open chromatin sites and maps transcription factor binding events in regulatory elements genome-wide, without needing any prior information about which proteins are bound. By correlating ATAC-seq patterns with other features, such as gene expression, it was possible to delineate the fine-scale architecture of the regulatory framework. Chromatin accessibility signatures were generated for each sample individually with detection of differential peaks between disease and control states to generate an initial possible disease-state signature.

Initial cohort: ATAC-seq was carried out as described (Milani et al., 2016)*.* Briefly, cells were lysed in cell lysis buffer (10  mM Tris-HCl, pH 7.4, 10  mM NaCl, 3 mM MgCl2, 0.1% IGEPAL CA-630, protease inhibitors) on ice for 5 min and centrifuged at 230 rcf for 5 min at 4°C. The pellet, containing the nuclei, was re-suspended in 25  μl of 1X Tagment DNA Buffer (Illumina). 50K nuclei were subjected to transposase reaction (Nextera - Illumina) followed by DNA purification. The tagmented DNA was PCR amplified using Nextera indexing primers (Illumina) and loaded on 2% agarose gel. Nucleosome-free fragment (175–250 bp) were size selected from the gel and further amplified by PCR to obtain the final libraries. The libraries were sequenced using the Illumina HiSeq 2000 platform (single end, 50 bp). All samples passed quality control checks that included morphological evaluation of nuclei, agarose gel electrophoresis of libraries, and real-time qPCR to assess the enrichment of open-chromatin sites. The quality of the sequencing was assessed using FastQC and the reads were aligned to GRCh37 genome build using BWA. Open chromatin regions were identified separately for each sample using the peak-calling software MACS2 (Zhang et al., 2008) and differentially open sites were determined using DEseq2 (FDR<0.1). Peaks were assigned to unique genes using the default HOMER (Heinz et al., 2010) parameters, and gene ontology analysis was performed using GOrilla (Eden et al., 2009).

#### Data integration

A hierarchical strategy was used for data integration. Transcriptional regulators were inferred from the combination of ATAC-seq and RNA-Seq data, and then connections were assessed among these transcriptional regulators and those detected directly by the proteomics.

**Inferring transcriptional regulators**: Accessible chromatin regions, identified by ATAC-seq, were combined with differential gene expression data to predict transcription factors (TFs) that contribute to differences in transcriptomics profiles between C9 and controls. Specifically, the union of peaks detected in ALS and control samples were used to identify peaks proximal (±2.5 kb) and distal (± 50kb) to gene transcription start sites (TSS), which were further divided into those with high and low CpG content. A normalized CpG metric was used as previously described (Soltis et al., 2017). The enrichment of known motifs was determined using HOMER. The analysis was performed separately for high and low CpG content peaks near (±10 kb or 50kb) differentially expressed genes as the foreground and corresponding regions near all known genes as the background.

#### Network analysis

Omics Integrator (Tuncbag et al., 2016) was used to search for previously reported protein-protein interactions that link proteins detected by mass spectrometry and the inferred transcription factors. Taking a network approach, proteins and TFs were represented as nodes and assigned prizes based on their experimental significance. Specifically, protein prizes were assigned according to the fold change between C9 and control samples and prizes for TFs were assigned according to false discovery rate (see above). These proteins were mapped on a network of physical interactions in which each edge was scored for reliability based on the underlying experimental data. The algorithm searches for disease-associated subnetworks that retain the maximum prizes while avoiding unreliable interactions which are formalized as the Prize-Collecting Steiner Forest problem. The aim is to find a forest solution $F\left(V_{F},E_{F}\right)\;$that maximizes the objective function:

$$f\left(F\right)=\beta \cdot \underset{v\in V_{F}}{\sum }p\left(v\right)-\underset{e\in E_{F}}{\sum }c^{\prime }\left(e\right)+\omega \cdot \kappa$$

The first term is the sum of prizes included in F, scaled by a model parameter β. The second term is a cost function which serves the purpose of only including a node in F if the objective function is minimized. The last term allows for the inclusion of κ trees by introducing a root node $v_{0}$ that is connected to every other node with a weight ω. This method not only performs feature selection by filtering out protein prizes that are expensive to connect, but also identifies “Steiner” proteins that were not detected as changing in the experiments, but are strongly implicated by the structure of the interactome. A Steiner node is typically included when its interaction neighbors are significant proteins identified from biological experiments. To avoid a bias toward proteins that have many known interactions (high-degree nodes), a regularization term on edges was imposed such that the cost of an edge between nodes a and b monotonically increases with $d_{a}$ and $\;d_{b}$, the node degrees of a and b:

$$c^{\prime }\left(e\right)=\;c\left(e\right)+\alpha \frac{d_{a}\cdot d_{b}}{\left(N-d_{a}-1\right)\left(N-d_{b}-1\right)+d_{a}\cdot d_{b}}.$$

This regularization term corresponds to the probability that an edge exists between a and b given the number of nodes in the interactome, N, and the degrees of a and b. c(e) is the cost of the edge which is inversely related to the amount of experimental evidence supporting the physical interaction between a and b given by iRefIndex (Razick et al., 2008). Finally, we acknowledge that the algorithm is susceptible to noise in the interactome, so we ran the experiment 100 times with randomly added noise to the interactome and chose the top 400 nodes that appeared most frequently and removed any disconnected nodes. Additionally, the specificity of the network was assessed by assigning the input prize values to random nodes in the interactome and measuring the frequency that each node appears. These experiments were repeated for a parameter grid and a network was selected that 1) performed feature selection (i.e., did not include the entire input prize list), 2) was specific (as determined by the calculations using randomly assigned prizes), and 3) had a degree distribution that matched that of the input prize file. As C9ORF72 was not detected in the proteomics measurements, C9ORF72 inclusion was forced in the network by artificially assigning it a large prize. Network nodes were then sorted by subcellular location based on the Compartments database (Binder et al., 2014) and plotted in Cytoscape.

#### Drosophila network analysis

The genes that were tested in the Drosophila model were categorized into three groups: causal, compensatory and non-contributory. For example, we reasoned that genes that were significantly upregulated in ALS and whose knock-down in fly suppressed or enhanced eye degeneration were likely causal or compensatory genes, respectively. Similarly, those that were significantly downregulated in ALS and were enhancers or suppressors of eye degenerations were likely causal or compensatory, respectively. Genes whose knock-down in the fly model showed little to no effect on eye degeneration were categorized as non-contributory.

Next, previously annotated directed interactions were used that originated from the ReactomeFiViz and KEGG databases (Kanehisa et al., 2017; Wu et al., 2014). The resulting directed network was composed of 9,336 nodes connected by 166,907 directed edges. For any two proteins that were labeled as either causal or compensatory, all directed paths of length at most 2 were identified. Only paths that were concordant with the data were considered, by not allowing paths:

1 to contain genes that are *not* expressed in iMNs. This was defined by taking the top 70% of expressed gene transcripts across all 7 iMNs lines.
2 whose predicted effect on protein activity is discordant with measured protein expression. For instance, if A->B, but A is up in ALS and B is down in ALS, this edge is excluded from further analysis. Direction of interaction (activating or inhibiting) was extracted from ReactomeFiViz and KEGG databases (Kanehisa et al., 2017; Wu et al., 2014) to determine the predicted effect.

### Quantification and statistical analysis

*Immunostaining:* The plots shown in Figure 1C are average results from quantified images of the respective immunostains in Figure 1B. The healthy control donors (CTR) comprised of n = 3 independent iPSC lines, while the C9-ALS comprised of n = 4 C9ORF72 repeat expansion donor iPSC lines. Total cells were quantified by nuclear staining with Hoechst 33258 in n = 9 sites across a culture well and percent positive cells for a respective marker were calculated for each site. Average positive marker expression was then calculated for each well. Each marker immunostain was performed across independent wells 3 times and respective average percent positive cells were obtained for each iPSC lines. All statistical analyses for percent SMI32, TuJ1, Map2a/b, GFAP and nestin levels were performed using an unpaired t test and the differences between CTR and C9-ALS groups were insignificant. Error bars represent standard error of the mean. Quantification of Nup98 spots. Student’s t test was used to calculate statistical significance (Gendron et al., 2017). *RNA-Seq*: Generalized linear models were used with negative binomial distribution to estimate fold change between ALS and control samples for each gene. Wald test was performed for hypothesis testing, which is a one-sided test. Sample size n for control and ALS was 3 and 4, respectively, in the first cohort and 4 and 6, respectively, in the second cohort. Significant DEGs and enrichment terms were chosen based on a 10% FDR. *Proteomics*: Throughout Trans Proteome Pipeline (TPP) and OpenSWATH, a 1% FDR cutoff was employed in identification of transitions/peptides and in OpenSWATH matching to the peptide library. MAP DIA (Teo et al., 2015) was used on MS2 normalized transition level data obtained from OpenSWATH. Transitions falling outside of 2 standard deviations were filtered out. An additional correlation filter of 0.2 was used to filter out any residual outliers. Intensities of the remaining transitions were summed for peptide, and then protein level quantification. Differential expression analysis of designated groups was performed by MAP DIA using analysis based on a Bayesian latent variable model with Markov random field prior. Output for differential expression included log2FC, confidence score, FDR and log(OddsofDifferentialExpression). Log2 fold changes were deemed significant if they had FDR at 1% or lower, a confidence score of .95 or above, a positive log(oddsDE) and an abs(log2FC) of .6 or above. For IPA, the 924 differentially expressed proteins and their corresponding log2FC values were used, with analysis settings for reference set: Ingenuity Knowledge Bases, direct relationships, using all data sources, experimentally observed interactions and filtered for human genes in primary tissues and human cell lines. For pathway enrichment analysis, GOrilla (Eden et al., 2009) was used. The DIA filtered list of 3742 proteins was used as the background list for analysis of target sets. A p-value threshold of 10^-3^ was used to determine enriched GO Biological Process terms. *ATAC-seq*: Differentially open sites were called using the DESEQ2 pipeline with FDR ≤ 0.1. *Data integration*: All GO enrichments were performed using a one-sided hypergeometric test implemented by GOrilla. Motif enrichments were calculated via HOMER, which searches for de novo motif matches that are enriched in a set of foreground sequences relative to a given set of background sequences using a one-sided hypergeometric test. Enrichment of ALS-associated genes was calculated using a one-sided hypergeometric implemented using the hypergeometric module in Scipy v0.14. Enrichments of genes between -omics assays were also calculated using a one-sided hypergeometric test implemented using the hypergeometric module in Scipy v0.14. For each pair of assays, the background was the set of genes that was detected in both assays. *Drosophila eye screen*: Flies were aged to 15 days after eclosion. 3 biological replicates were carried out per cross. 15 females flies were scored per cross. The average score of these 15 flies was taken as the average for one biological replicate. The average of all 3 biological replicates rounded to the nearest 0.5 of a point was used for the final rounded rough eye score

### Additional resources

The NIH LINCS portal: http://lincsportal.ccs.miami.edu/dcic-portal/.

The NeuroLINCS data portal: https://lincsproject.org/.

The AnswerALS data portal: https://dataportal.answerals.org/home.

## Data Availability

• ATAC-seq and RNA-Seq raw data is available in dbGAP (https://www.ncbi.nlm.nih.gov/gap) and processed data is available through the LINCS portal (http://lincsportal.ccs.miami.edu/dcic-portal/). Proteomics raw data is available in the CHORUS database (https://chorusproject.org/pages/dashboard.html#/search/Neurolinc/projects) and processed data is available through the LINCS portal (http://lincsportal.ccs.miami.edu/dcic-portal/). Replication cohort raw and processed data, and all other datasets described above are also currently publicly available through the AnswerALS data portal (https://dataportal.answerals.org/home). • All data and code are available from the corresponding authors upon reasonable request. • Any additional information required to reanalyze the data reported in this paper is available from the lead contact upon request.

## Supplemental information

Document S1. Figures S1–S18 and Tables S1–S8

Table S9. List of genes analyzed for statistically significant differences from RNA-Seq, see Table S6

Table S10. Summary of proteomics, see Table S7 and corresponding tabs

Table S11. Summary of pathogenic (P) and likely pathogenic (LP) variants called by Intervar117 that are less than 20% frequent within the population

Table S12. Genes used for linear regression analysis of gene variant and expression comparisons

Table S13. List of ALS-associated genes from text mining sources and experimental sources

Table S14. Summary table of RNAi lines used and full G4C2 fly screen results

Table S15. Proteomics fold changes (ALS/CTR) and false discovery rates (FDR) from the original and validation experiments for proteins in the fly network (Figure 5)
