# Supplementary material for: An integrated multi-omic analysis of iPSC-derived motor neurons from C9ORF72 ALS patients
Source: iScience. 2021 Oct 12;24(11):103221. doi: 10.1016/j.isci.2021.103221 (PMC8554488; doi:10.1016/j.isci.2021.103221)
Supplement: Document S1. Figures S1–S18 and Tables S1–S8 [file mmc1.pdf]

iScience, Volume *24*

Supplemental information

An integrated multi-omic analysis of iPSC-derived motor neurons from C9ORF72 ALS patients

The NeuroLINCS Consortium, Jonathan Li, Ryan G. Lim, Julia A. Kaye, Victoria Dardov, Alyssa N. Coyne, Jie Wu, Pamela Milani, Andrew Cheng, Terri G. Thompson, Loren Ornelas, Aaron Frank, Miriam Adam, Maria G. Banuelos, Malcolm Casale, Veerle Cox, Renan Escalante-Chong, J. Gavin Daigle, Emilda Gomez, Lindsey Hayes, Ronald Holewenski, Susan Lei, Alex Lenail, Leandro Lima, Berhan Mandefro, Andrea Matlock, Lindsay Panther, Natasha Leanna Patel-Murray, Jacqueline Pham, Divya Ramamoorthy, Karen Sachs, Brandon Shelley, Jennifer Stocksdale, Hannah Trost, Mark Wilhelm, Vidya Venkatraman, Brook T. Wassie, Stacia Wyman, Stephanie Yang, NYGC ALS Consortium, Jennifer E. Van Eyk, Thomas E. Lloyd, Steven Finkbeiner, Ernest Fraenkel, Jeffrey D. Rothstein, Dhruv Sareen, Clive N. Svendsen, and Leslie M. Thompson

Supplemental Figure Legends

Figure S1: Related to Figure 1: Schematic for generation of iPSC-motor neurons. (A) iPSC-derived motor neurons precursor spheres (iMPS) and (B) iMPS-derived motor neurons (iMNs), and media components for each stage.

Figure S2: Related to Figure 1 and STAR Methods: Replication cohort differentiations. (A) Schematic for generation of the replication cohort of CTR and C9-ALS iPSCs into direct iPSC-derived motor neurons (diMNs) cultures using a rapid 3 stage protocol used by NeuroLINCS for transcriptomics, proteomics and ATAC-seq assays. (B) Violin plots quantifying levels of NEFH (SMI32), Islet1, Nkx6.1, and TuJ1 in control and C9-ALS diMNs cultures. Percent ISLET1-positive cell count in CTR vs C9-ALS groups as statistically significant (***). Two-tailed p-value = 0.0009; Unpaired t test with Welch's correction. CTR n=7 and C9-ALS n=6.

Figure S3: Figure S3, Related to Figure 1 and STAR Methods: Representative images of diMNs from the replication cohort. Images show distribution of neural cell populations from 7 control and 6 C9-ALS iPSCs marked by SMI32 (NEFH), Islet1, Nkx6.1, and TuJ1 (TUBB3) in control and C9-ALS diMNs cultures. For each set of stains and for every cell line differentiated into diMNs, there is dotted box in the main image which shows the region that is magnified in the adjacent image. All scale bars are 100 μm.

Figure S4: Figure S4, Related to Figure 1 and STAR methods: G-band karyotype analysis of iPSCs from first cohort. G-band karyotypes depict normal cytogenetic profiles in the 3 control iPSC lines and the 4 C9-ALS iPSC lines used in this study. (p, passage at which cells were harvested).

Figure S5, Related to Figure 1 and STAR methods: G-band karyotype analysis of iPSCs from replication cohort. G-band karyotypes depict normal cytogenetic profiles in the replication cohort of the additional 7 C9-ALS and the 6 control iPSC lines used in this study. (P, passage at which cells were harvested).

Figure S6, Related to Figure 1 and STAR methods: DNA fingerprinting of iPSCs and iMNs from first cohort. DNA Fingerprinting human 9 species-specific short-tandem repeat (STR) marker profiling confirms that the reprogrammed iPSCs and the differentiated iMNs used for 'OMICS assays in this study match the parental donor fibroblasts. *N/A (not applicable) as the parent fibroblast line was not available for comparison purposes. The genetic profile for the sample was compared to the cell line genetic profiles available in the DSMZ STR database and to all previously submitted profiles in the Cedars-Sinai iPSC Core. The profiles were found to be unique and did not match to any previously submitted profiles. The genetic profile established for this sample can be used for future comparisons for this cell line.

Figure S7, Related to Figure 1 and STAR methods: DNA fingerprinting of iPSCs and iMNs from replication cohort. DNA Fingerprinting human 9 species-specific STR marker profiling confirms that the replication cohort of the reprogrammed iPSCs and the differentiated diMNs used for 'OMICS' assays in this study match the parental donor PBMCs and iPSCs. * N/A (not applicable) as the parent PBMCs were not available for comparison purposes. The genetic profile for the sample was compared to the cell line genetic profiles available in the DSMZ STR database and to all previously submitted profiles in the Cedars-Sinai iPSC Core. The profiles were found to be unique and did not match to any previously submitted profiles. The genetic profile established for this sample can be used for future comparisons for this cell line.

Figure S8, Related to Figure 2: PCAs to show gene expression variance between samples used in the first and second cohorts. (A) PCA using the top 500 highly variable genes (HVGs) for the first cohort and (B) PCA using the top 500 HVGs for the second cohort. Some separation can be seen between ALS and control in A along PC1 with no clear separation between the 2 groups in B.

Figure S9, Related to Figure 2: RNAseq data visualization, cell types and pathways. (A) Volcano plots of log2 fold change and -log2(adjusted pvalue) from RNA-Seq datafor original (iMN) and replication (DE4 = d18 dIMN) cohorts. (B) MA plots of log2 fold change and mean normalized counts from RNA-Seq data for original (iMN) and replication (DE4) cohorts. (C) Cell type-specific analysis of 828 DEGs from the revealing an enrichment for cortical and motor neurons. For each cell type, the size of the hexagon is scaled to the number of specific genes at different stringency thresholds. (D) NRG1 subnetwork showing upregulated target genes (Red) from C9 vs Control iMN (original cohort) and predicted activation of upstream regulators (Orange). (E) Fold change levels of MMPs and associated substrates in C9 vs control iMNs (original cohort). (F) Percent alternative splicing types in C9 iMNs (original cohort) (G-I) GO enrichment analysis of significant differentially spliced genes.

Figure S10, Replated to Figure 2: Proteomics data QC. (A) Mass spectrometry (MS) runs result in 3844 unique hits, based on 23,436 peptides. Shared hits are indicative of peptides that mapped to multiple proteins; these were not used in further data analysis. (B) Total ion current distribution for each file shows similar levels of MS1 and MS2 TIC. (C) Log2 Protein Intensity distribution of MS2 normalized protein data shows the spread of protein intensities for each MS run. There are no major differences between sample distribution and no major difference in intensities between control and disease samples. (D) ALS and Control normalized protein intensity show a high correlation, with R = .9660. This shows that both ALS and Control sample differentiations yielded

similar samples. (E) Correlation between overlapping differentially expressed proteins and genes is high. (F) Principal component analysis shows separation of ALS and Control in PC1 when PC1 and PC4 are mapped. (G) IPA shows predicted inhibition of RNA processing and splicing.

Figure S11, Related to Figure 2: ATACseq quality control and analysis. (A) A histogram of differential and consensus ATAC-seq peaks' distance to their nearest genes show that differential peaks lie further away from genes on average. (B) Consensus (left) and differential (right) peaks were mapped to Gencode annotations. A smaller proportion of differential peaks lie in promoters than consensus peaks. (C) Roughly 6% of all consensus peaks are open in ALS, and another 6% are open in control. The two sets of tracks on the right are examples from both of these categories. (D) Each differential peak was assigned to the nearest gene within 50kb and the number of ALS and CTR peaks were counted for each gene. The heatmap intensity indicates the number of genes with a given combination of ALS and CTR peak counts.

Figure S12, Related to Figure 2 and STAR methods: eQTLs in integrated network. Boxplots showing each significant genotype-gene expression comparison of genes also found in our integrated network. Y-axis: gene expression, X-axis: genotype. Genotype (Red = Ref, Green = Het, Blue = Homo). Dots are individual replicates of each sample in study (ALS= Red, Control = Blue).

Figure S13, Related to Figure 2 and STAR methods: eQTLs compared to known brain eQTLs. Boxplots showing all genotype-gene expression comparisons for known brain eQTLs found in our WGS and also found in our significant DEGs. Y-axis: gene expression, X-axis: genotype. Genotype (Red = Ref, Green = Het, Blue = Homo). Dots are individual replicates of each sample in study (ALS= Red, Control = Blue). Variant rsID are listed below each plot.

Figure S14, Related to Figure 2: Top GO enrichments for each assay.

Figure S15, Related to Figure 4: SUMO Subnetwork of disease network. Subnetwork from Figure 4C explores possible effects of decreased Sumoylation in C9-ALS lines.

Figure S16, Related to Figure 2 and STAR methods: Comparison to postmortem cervical spine data. Density and histogram plots from randomization tests. (A) Density plot of -log(p values) showing significance of overlap between 100 network randomizations and DEGs from ALS vs CTR postmortem cervical spine (FDR < 0.1), calculated by Fisher's Exact test. Dashed orange line marks -log(p value) of true integrated network (9.18E-05) compared to the postmortem DEGs. (B) Density plot of number of overlapping genes between 100 network randomizations and DEGs from ALS vs CTR postmortem cervical spine (FDR < 0.1). Dashed orange line marks the number of overlapping genes from the true integrated network (81 genes) compared to the postmortem DEGs. (C) Histogram and distribution of the numbers of DEGs found using 1000 randomized permutations. Red line marks value generated using actual sample labels. (D) Histogram and distribution of overlap between 1000 randomized DEG lists and our ECM subnet. Red line marks value generated using actual sample labels.

Figure S17, Related to Figure 5: Comparison of protein expression and eye phenotype. Density plot showing change in eye phenotype and change in protein expression for each fly perturbation shows no correlation.

Figure S18, Related to Figure 5 and 6: Comparison of proteins from the integrated network between two sets of cultured motor neuron experiments. The horizontal and vertical components of the arrows indicate protein fold changes (ALS/CTR) between the original (Figure 5c,d) and validation experiment, respectively. Arrows colored black indicate proteins whose fold

changes were in the same directions (consistent) between experiments. Arrows colored red indicate proteins whose fold changes were in different directions (inconsistent) between experiments. Arrows colored gray indicate nodes that were not detected in either experiment.

Supplementary Figure 1: Schematic for generation of iPSC-motor neurons.

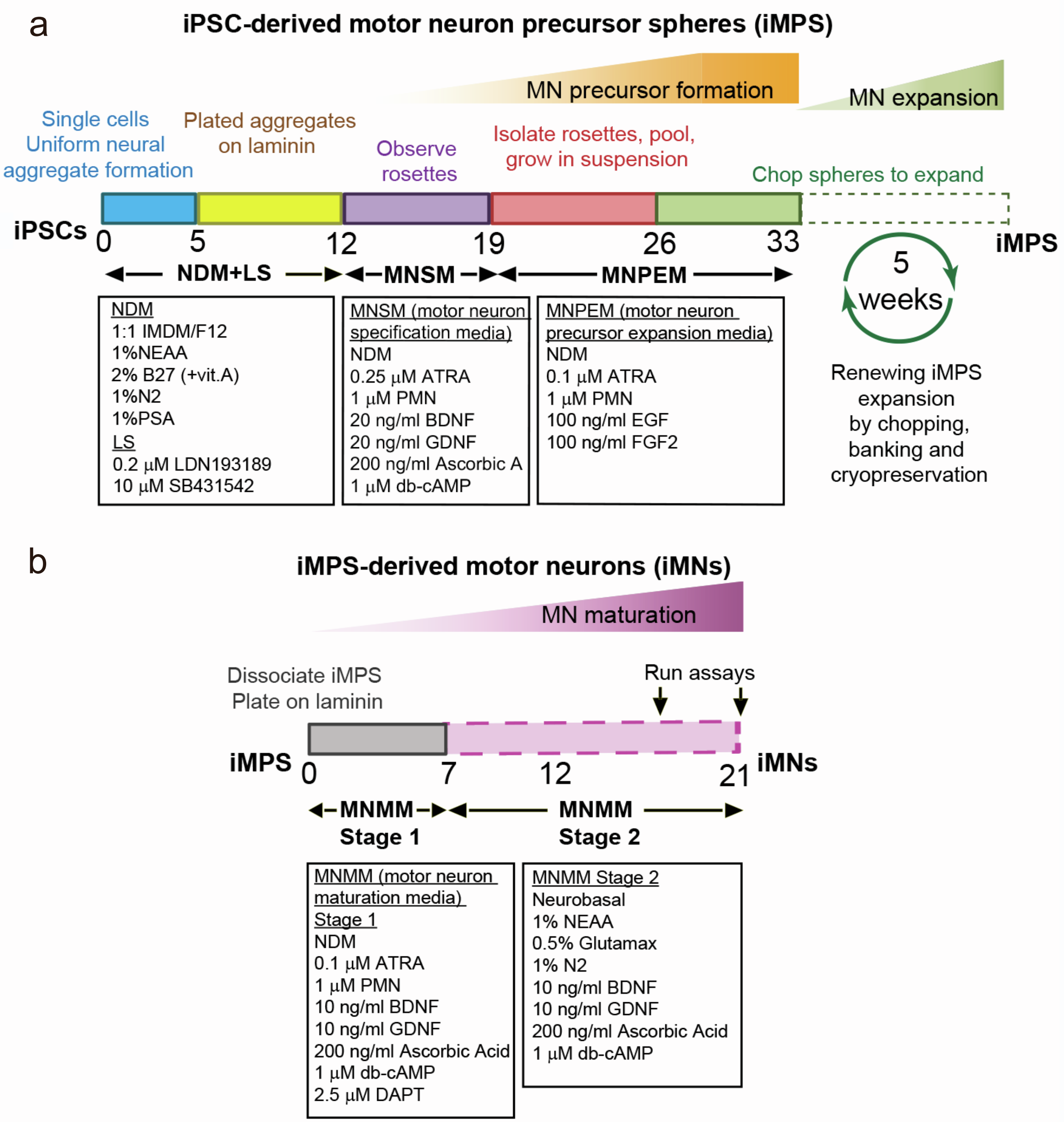

Supplementary Figure 2: Replication cohort differentiations.

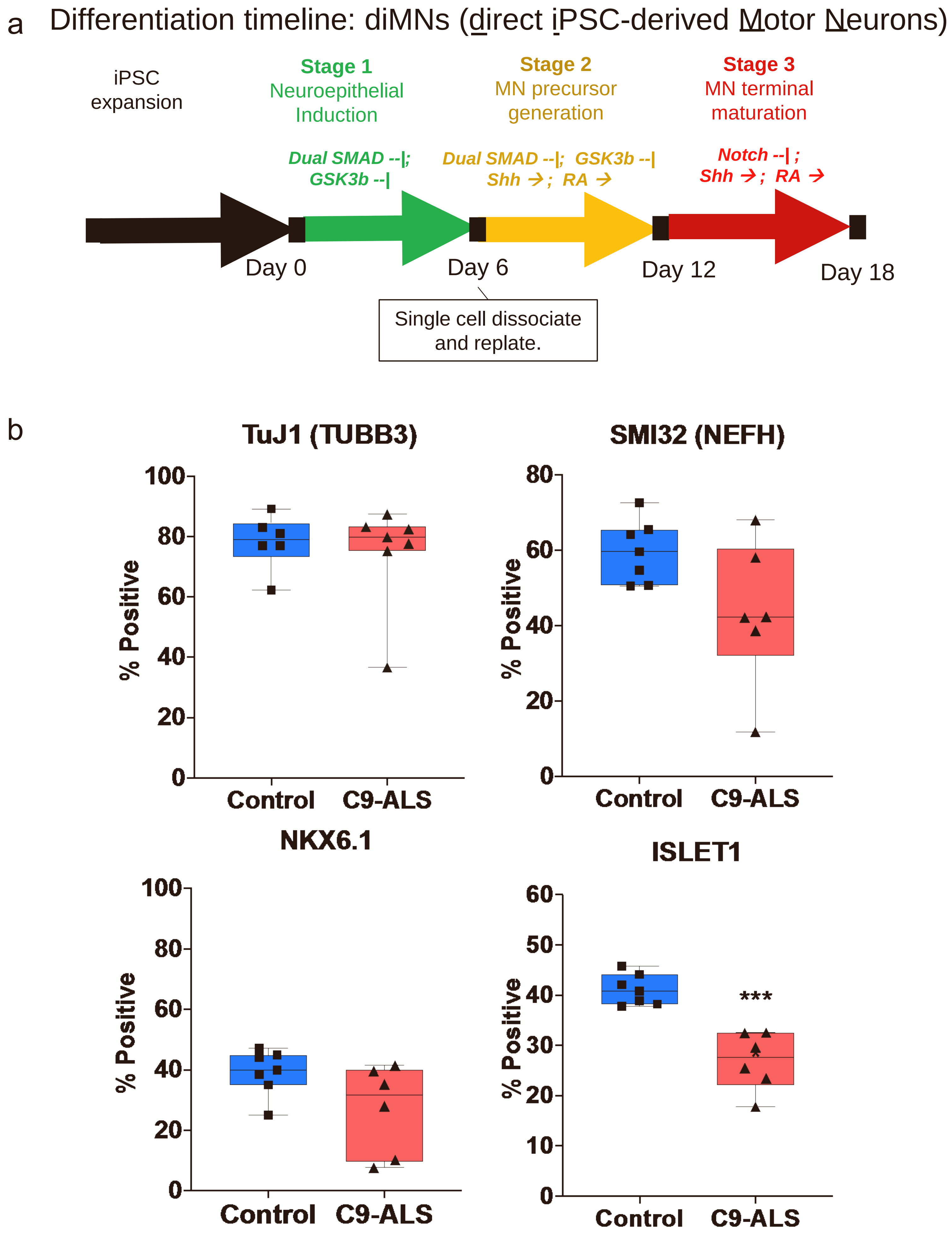

Supplementary Figure 3: Representative images of diMNs from the replication cohort.

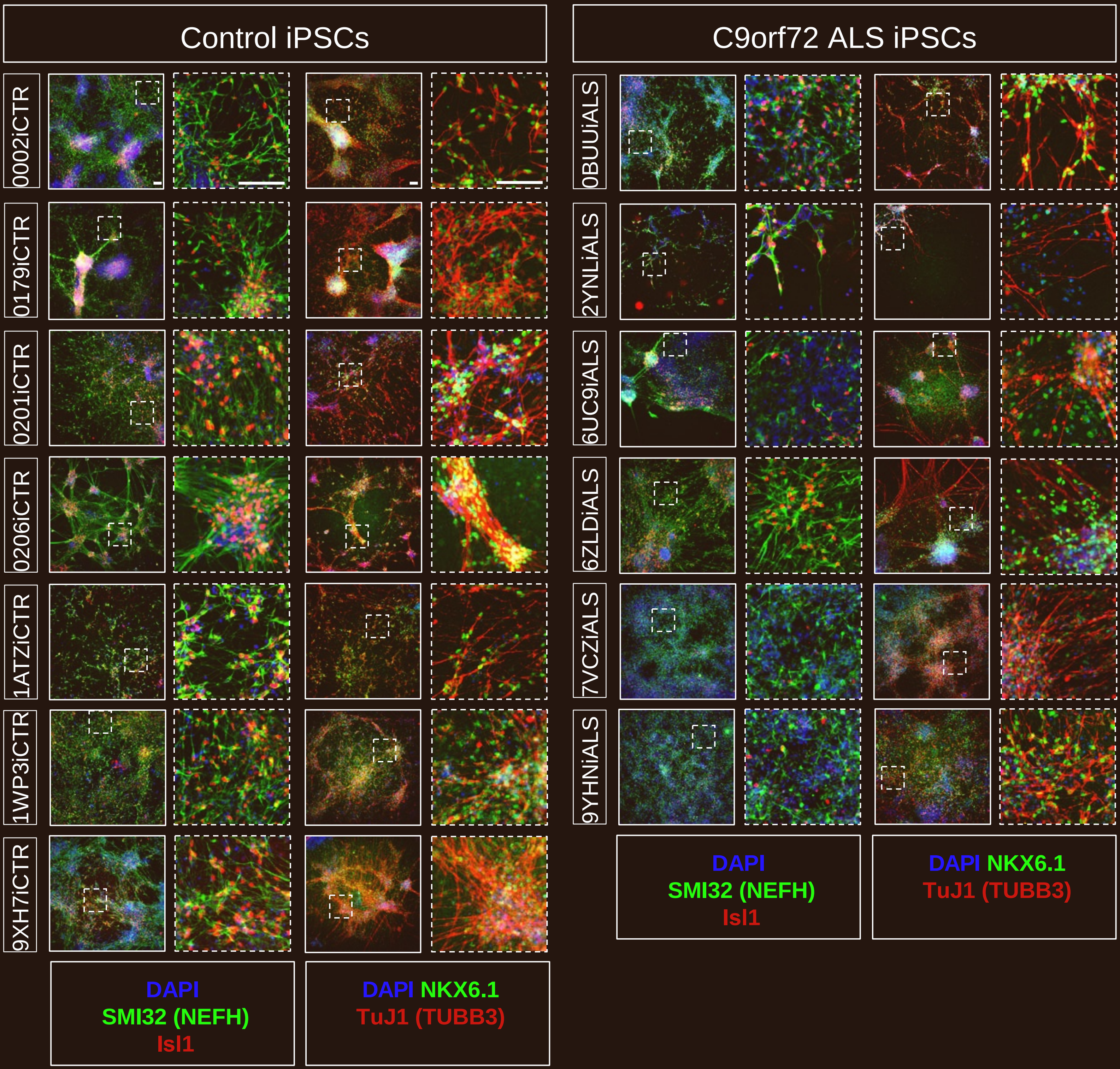

Supplementary Figure 4: G-band karyotype analysis of iPSCs from first cohort.

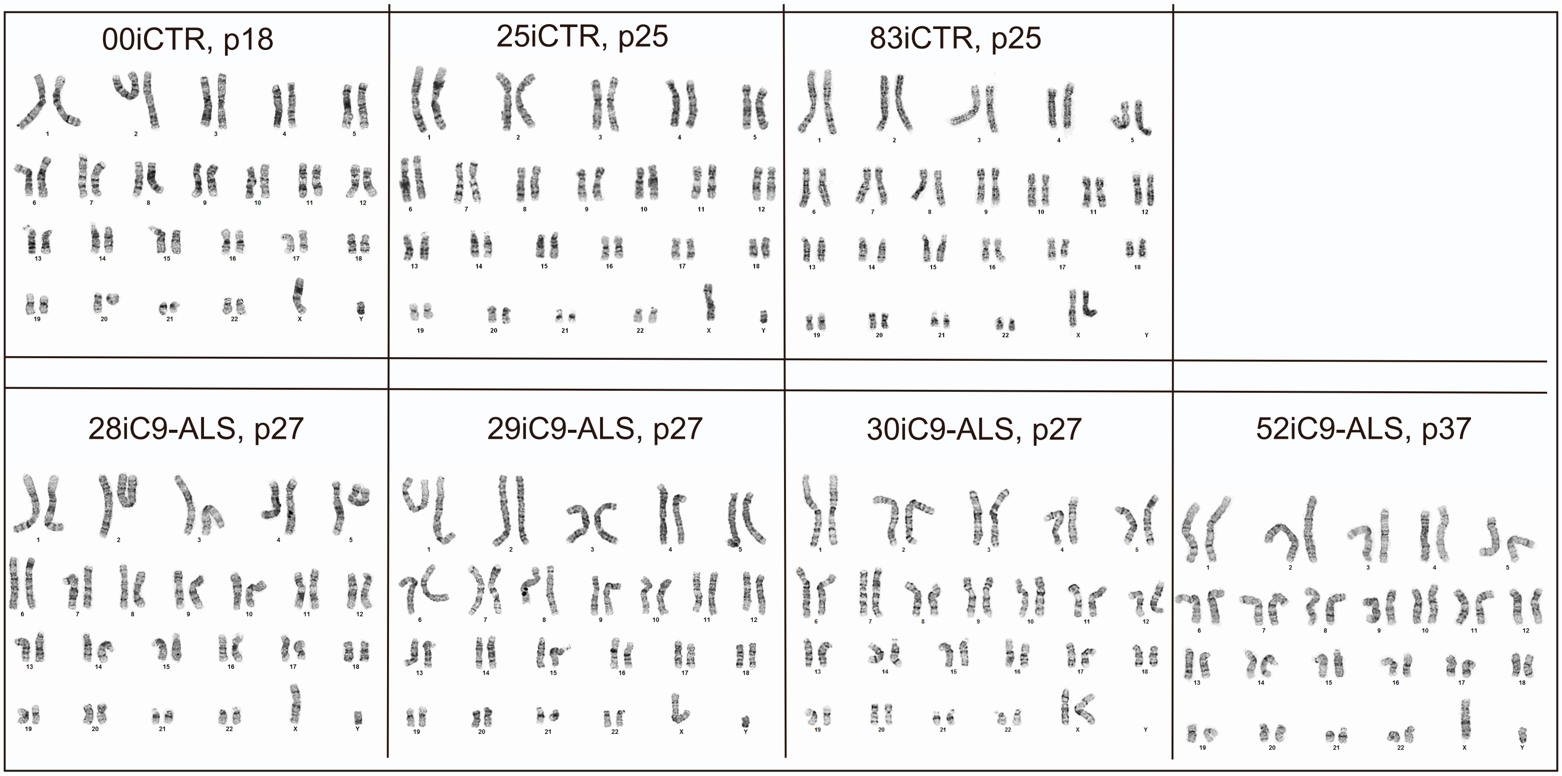

Supplementary Figure 5, G-band karyotype analysis of iPSCs from replication cohort.

CS0BUUiALS-n3, P19	CS2YNLiALS-n1, P18	CS6UC9iALS-n1, P19	CS6ZLDiALS-n1, P18
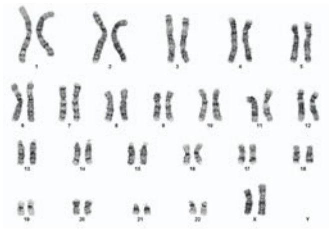	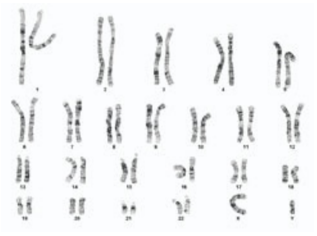	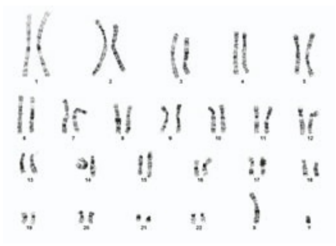	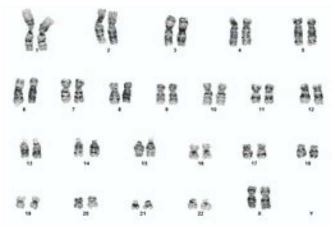
CS7VCZiALS-n3, P19	CS9YHNiALS-n1, P18		
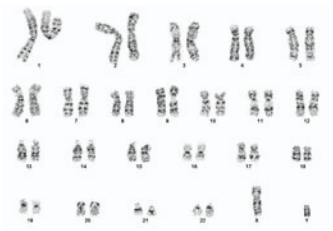	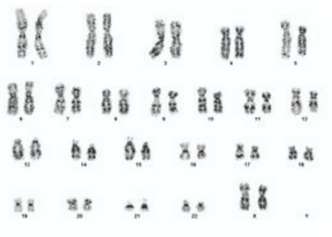		
CS0201iCTR-n4, P19	CS0206iCTR-n5, P20	CS1ATZiCTR-n2, P18	CS1WP3iCTR-n8, P20
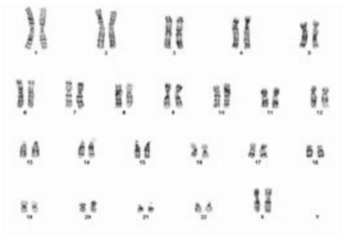	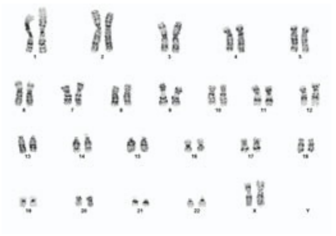	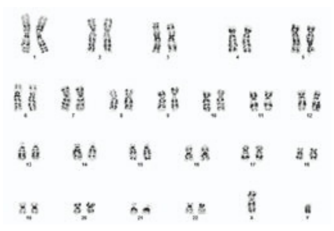	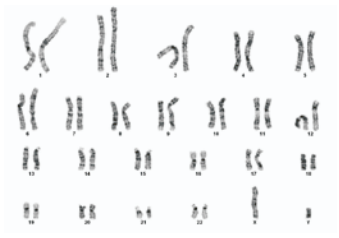
CS9XH7iCTR-n4, P21	CS0002iCTR-n1, P21	CS0179iCTR-n1, P22	
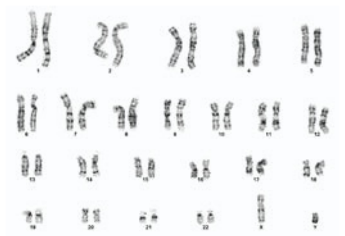	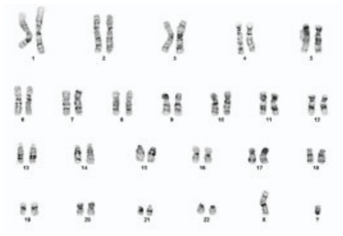	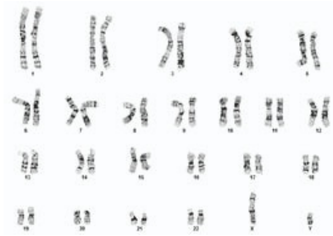	

Supplementary Figure 6: DNA fingerprinting of iPSCs and iMNs from first cohort.

Cell Line	Cell Type	AMEL	CSF1PO	D13S317	D16S539	D5S818	D7S820	TH01	TPOX	vWA	% Match
		Marker Name									
GM05400	Parent Line	X, Y	8, 13	11, 12	11, 12	8, 12	8, 9	6, 8	9, 11	16, 17	-
00iCTR	iPSC	X, Y	8, 13	11, 12	11, 12	8, 12	8, 9	6, 8	9, 11	16, 17	100%
00iCTR	iMN	X, Y	8, 13	11, 12	11, 12	8, 12	8, 9	6, 8	9, 11	16, 17	100%
ND30625	Parent Line	X, Y	12	12, 13	11, 12	11, 12	9, 10	9.3	8	18	-%
25iCTR	iPSC	X, Y	12	12, 13	11, 12	11, 12	9, 10	9.3	8	18	100%
25iCTR	iMN	X, Y	12	12, 13	11, 12	11, 12	9, 10	9.3	8	18	100%
GM02183	Parent Line	X	12	14	11	11, 12	8, 12	9, 9.3	8	16, 19	-
83iCTR	iPSC	X	12	14	11	11, 12	8, 12	9, 9.3	8	16, 19	100%
83iCTR	iMN	X	12	14	11	11, 12	8, 12	9, 9.3	8	16, 19	100%
F09128 *	Parent Line	N/A	N/A	N/A	N/A	N/A	N/A	N/A	N/A	N/A	-
28iAL	iPSC	X, Y	12	11, 12	10, 12	11, 12	10, 12	6, 8	8, 9	16, 17	-
28iALS	iMN	X, Y	12	11, 12	10, 12	11, 12	10, 12	6, 8	8, 9	16, 17	100%
F09229	Parent Line	X, Y	11, 13	11, 14	11, 12	11, 12	11, 12	7, 9	8, 9	15, 17	-
29iALS	iPSC	X, Y	11, 13	11, 14	11, 12	11, 12	11, 12	7, 9	8, 9	15, 17	100%
29iALS	iMN	X, Y	11, 13	11, 14	11, 12	11, 12	11, 12	7, 9	8, 9	15, 17	100%
F10-330 *	Parent Line	N/A	N/A	N/A	N/A	N/A	N/A	N/A	N/A	N/A	-
30iALS	iPSC	X	12	9, 14	11, 12	13	10	7, 9.3	8	16, 17	-
30iALS	iMN	X	12	9, 14	11, 12	13	10	7, 9.3	8	16, 17	100%
F09152	Parent Line	X, Y	10, 11	8	9, 11	12	9, 10	8, 9.3	8, 11	16, 17	-
52iALS	iPSC	X, Y	10, 11	8	9, 11	12	9, 10	8, 9.3	8, 11	16, 17	100%
52iALS	iMN	X, Y	10, 11	8	9, 11	12	9, 10	8, 9.3	8, 11	16, 17	100%

Supplementary Figure 7, DNA fingerprinting of iPSCs and iMNs from replication cohort.

Cell Line	Cell Type	AMEL	CSF1PO	D13S317	D16S539	D5S818	D7S820	TH01	TPOX	vWA	% Match
NEUEM720BUU	PBMC	X	10	11,12	12	12,13	11,12	6	8	15,18	-
CS0BUUiALS	iPSC	X	10	11,12	12	12,13	11,12	6	8	15,18	100%
CS0BUUiALS	diMNs	X	10	11,12	12	12,13	11,12	6	8	15,18	100%
NEUVX902YNL	PBMC	-	-	-	-	-	-	-	-	-	-
CS2YNLiALS	iPSC	X,Y	12,13	12,14	10,11	11	8,11	6	8	16,18	-
CS2YNLiALS	diMNs	X,Y	12,13	12,14	10,11	11	8,11	6	8	16,18	100%
NEUUL256UC9	PBMC	X,Y	12	8	13	12	10,12	6,9.3	8	18,20	-
CS6UC9iALS	iPSC	X,Y	12	8	13	12	10,12	6,9.3	8	18,20	100%
CS6UC9iALS	diMNs	X,Y	12	8	13	12	10,12	6,9.3	8	18,20	100%
NEUPK546ZLD	PBMC	X	12,13	8,12	11,12	10,12	12	9.3	11	16,18	-
CS6ZLDiALS	iPSC	X	12,13	8,12	11,12	10,12	12	9.3	11	16,18	100%
CS6ZLDiALS	diMNs	X	12,13	8,12	11,12	10,12	12	9.3	11	16,18	100%
NEUFV237VCZ	PBMC	X,Y	10	11,12	11	11	9,10	9.3	8,11	19	-
CS7VCZiALS	iPSC	X,Y	10	11,12	11	11	9,10	9.3	8,11	19	100%
CS7VCZiALS	diMNs	X,Y	10	11,12	11	11	9,10	9.3	8,11	19	100%
NEUDT709YHN	PBMC	X	12	8,12	11,13	11,12	9,10	7,9	10,12	17,20	-
CS9YHNiALS	iPSC	X	12	8,12	11,13	11,12	9,10	7,9	10,12	17,20	100%
CS9YHNiALS	diMNs	X	12	8,12	11,13	11,12	9,10	7,9	10,12	17,20	100%
W15-C201	PBMC	X	10,11	11,12	9,11	11,14	8,11	9,9.3	8	15,18	-
CS0201iCTR	iPSC	X	10,11	11,12	9,11	11,14	8,11	9,9.3	8	15,18	100%
CS0201iCTR	diMNs	X	10,11	11,12	9,11	11,14	8,11	9,9.3	8	15,18	100%
W15-C206	PBMC	X	10,12	12	11,13	11,12	9,10	9,9.3	8,11	14,17	-
CS0206iCTR	iPSC	X	10,12	12	11,13	11,12	9,10	9,9.3	8,11	14,17	100%
CS0206iCTR	diMNs	X	10,12	12	11,13	11,12	9,10	9,9.3	8,11	14,17	100%
NEUMN061ATZ	PBMC	X,Y	12,13	12	11,12	10,13	10,11	9, 9.3	8	14,19	-
CS1ATZiCTR	iPSC	X,Y	12,13	12	11,12	10,13	10,11	9, 9.3	8	14,19	100%
CS1ATZiCTR	diMNs	X,Y	12,13	12	11,12	10,13	10,11	9, 9.3	8	14,19	100%
NEUVW301WP3	PBMC	X,Y	10,12	8,12	9,11	12	8,9	6,7	9,11	17,18	-
CS1WP3iCTR	iPSC	X,Y	10,12	8,12	9,11	12	8,9	6,7	9,11	17,18	100%
CS1WP3iCTR	diMNs	X,Y	10,12	8,12	9,11	12	8,9	6,7	9,11	17,18	100%
NEUPW469XH7	PBMC	X,Y	11,12	12,14	11,12	12,13	8,11	9,9.3	8	15,18	-
CS9XH7iCTR	iPSC	X,Y	11,12	12,14	11,12	12,13	8,11	9,9.3	8	15,18	100%
CS9XH7iCTR	diMNs	X,Y	11,12	12,14	11,12	12,13	8,11	9,9.3	8	15,18	100%
CS-002	PBMC	X,Y	12	9,11	9,10	11,12	10,11	6,9.3	8,11	14,16	-
CS0002iCTR	iPSC	X,Y	12	9,11	9,10	11,12	10,11	6,9.3	8,11	14,16	100%
CS0002iCTR	diMNs	X,Y	12	9,11	9,10	11,12	10,11	6,9.3	8,11	14,16	100%
W14-C179	PBMC	X,Y	10,12	11	11	10,11	10,13	6,9.3	8	17,19	-
CS0179iCTR	iPSC	X,Y	10,12	11	11	10,11	10,13	6,9.3	8	17,19	100%
CS0179iCTR	diMNs	X,Y	10,12	11	11	10,11	10,13	6,9.3	8	17,19	100%

Supplementary Figure 8: PCAs to show gene expression variance between samples used in the first and second cohorts.

a

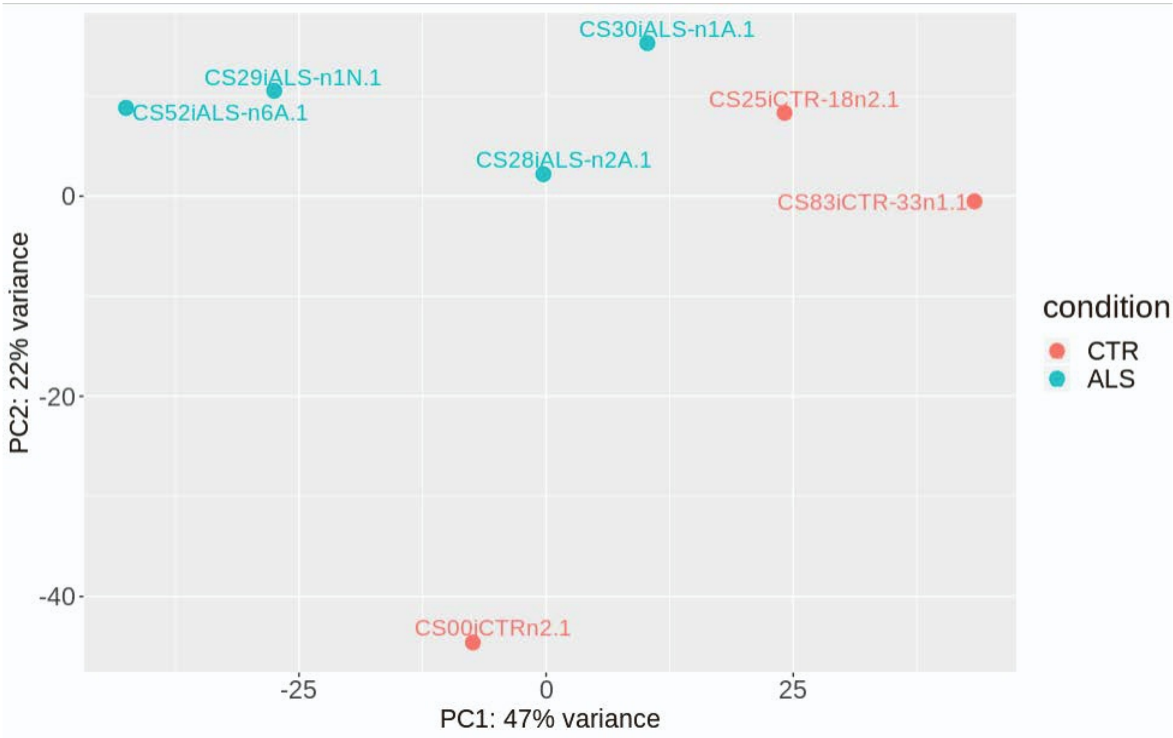

b

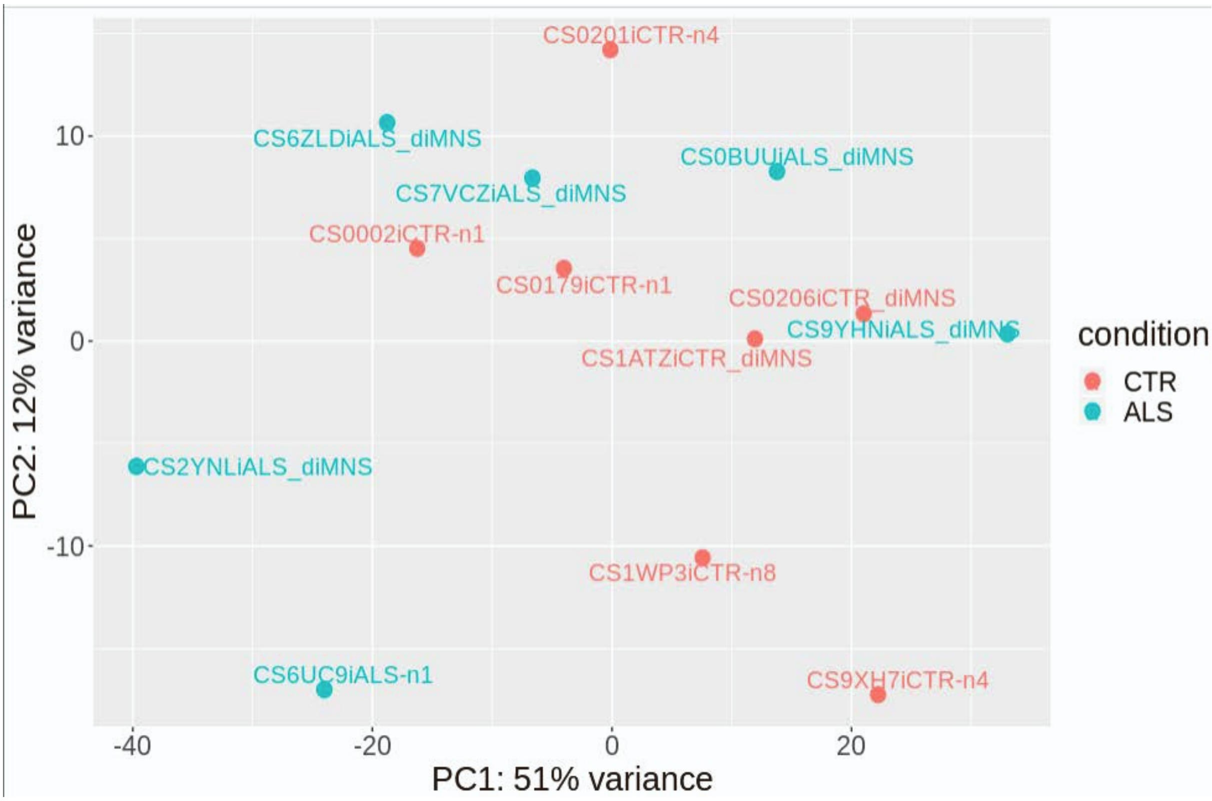

Supplementary Figure 9: RNAseq data visualization, cell types and pathways.

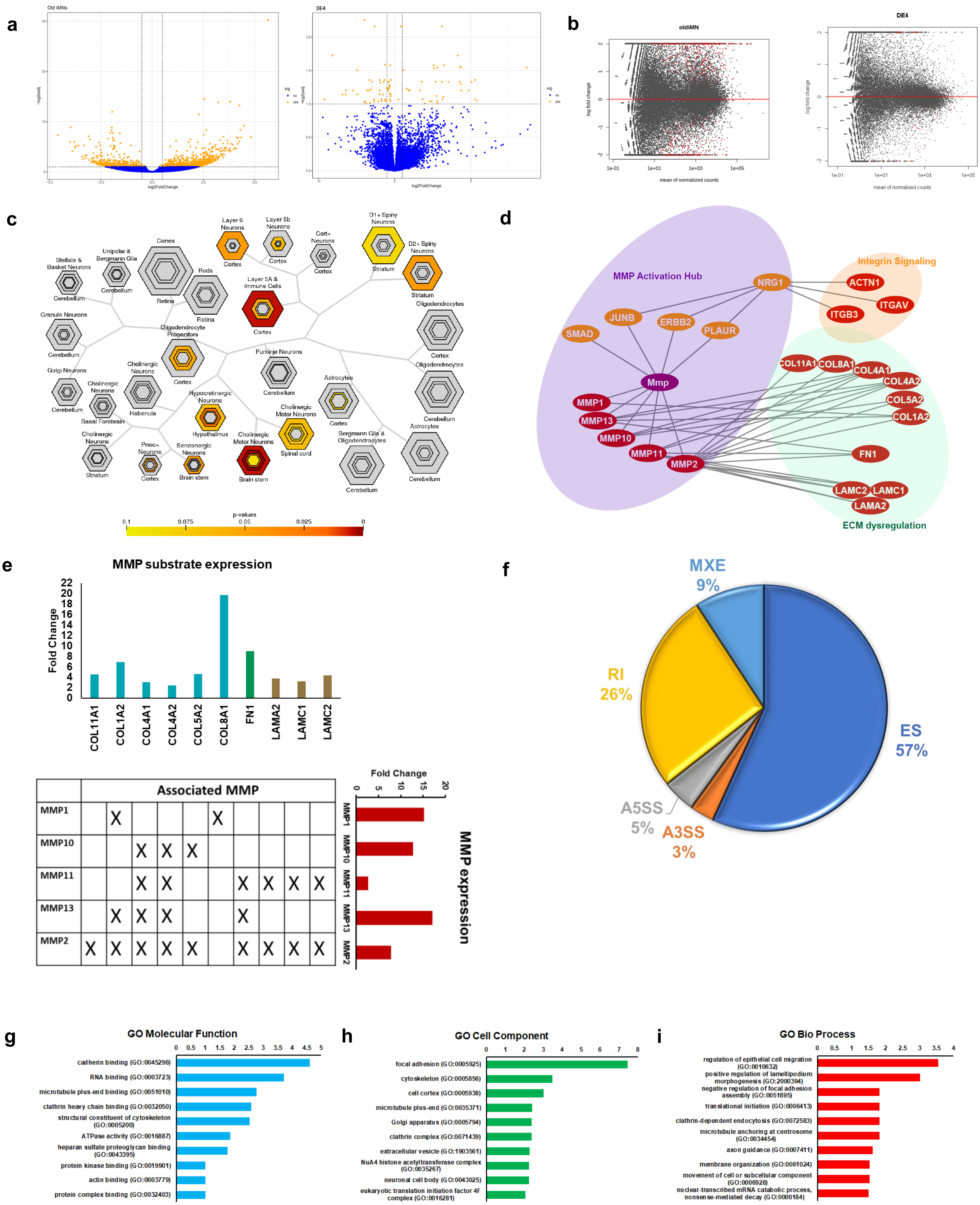

	Associated MMP									
MMP1		X				X				
MMP10			X	X	X					
MMP11			X	X			X	X	X	X
MMP13		X	X	X			X			
MMP2	X	X	X	X	X		X	X	X	X

Supplementary Figure 10: Proteomics data QC.

a

File Level Summary	MS Runs	Proteins	Peptides	Transitions
UniqueHits	15	3844	23436	160686
SharedHits	15	5518	26102	179580

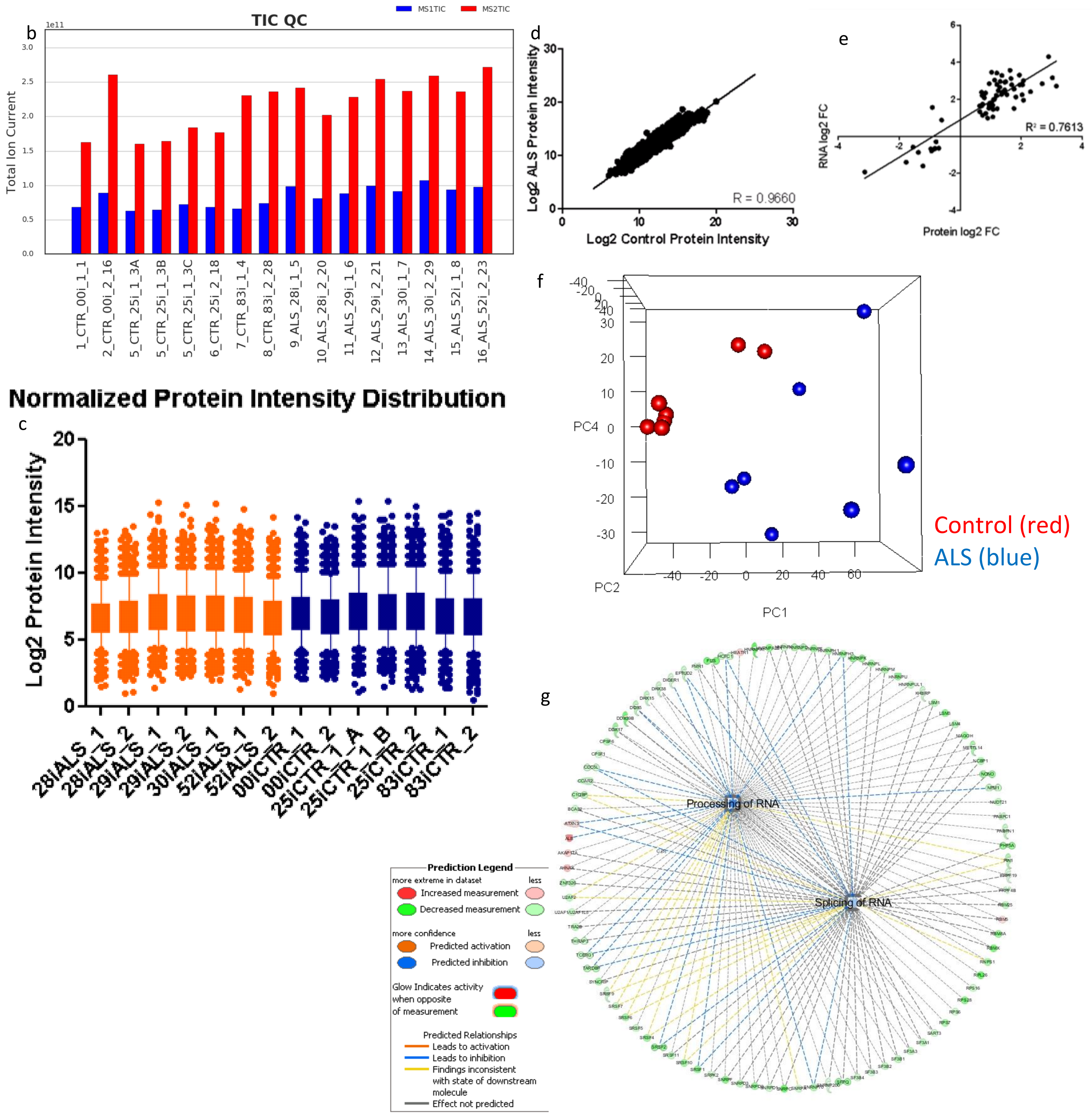

Supplementary Figure 11: ATACseq quality control and analysis.

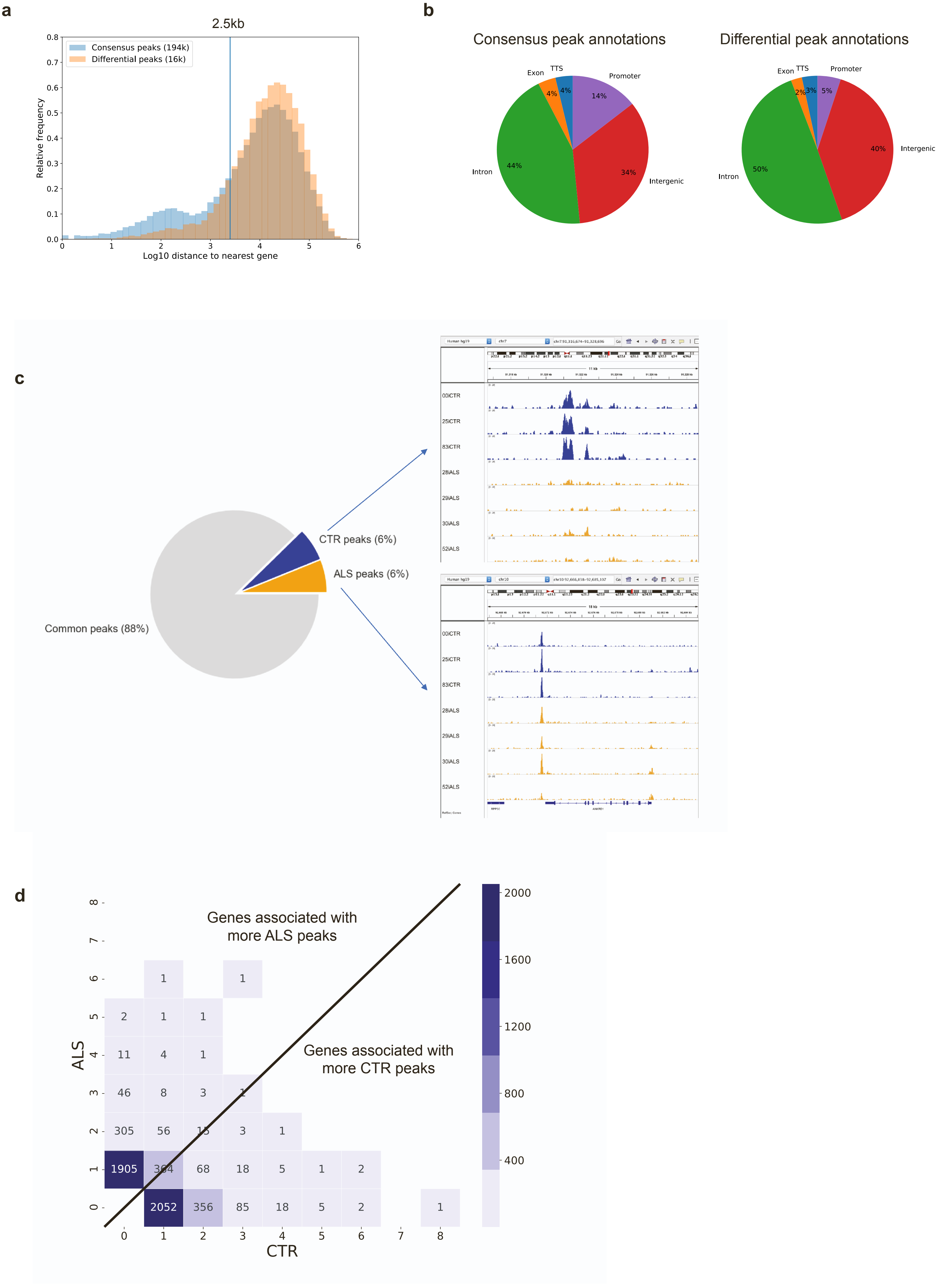

Supplementary Figure 12: eQTLs in integrated network.

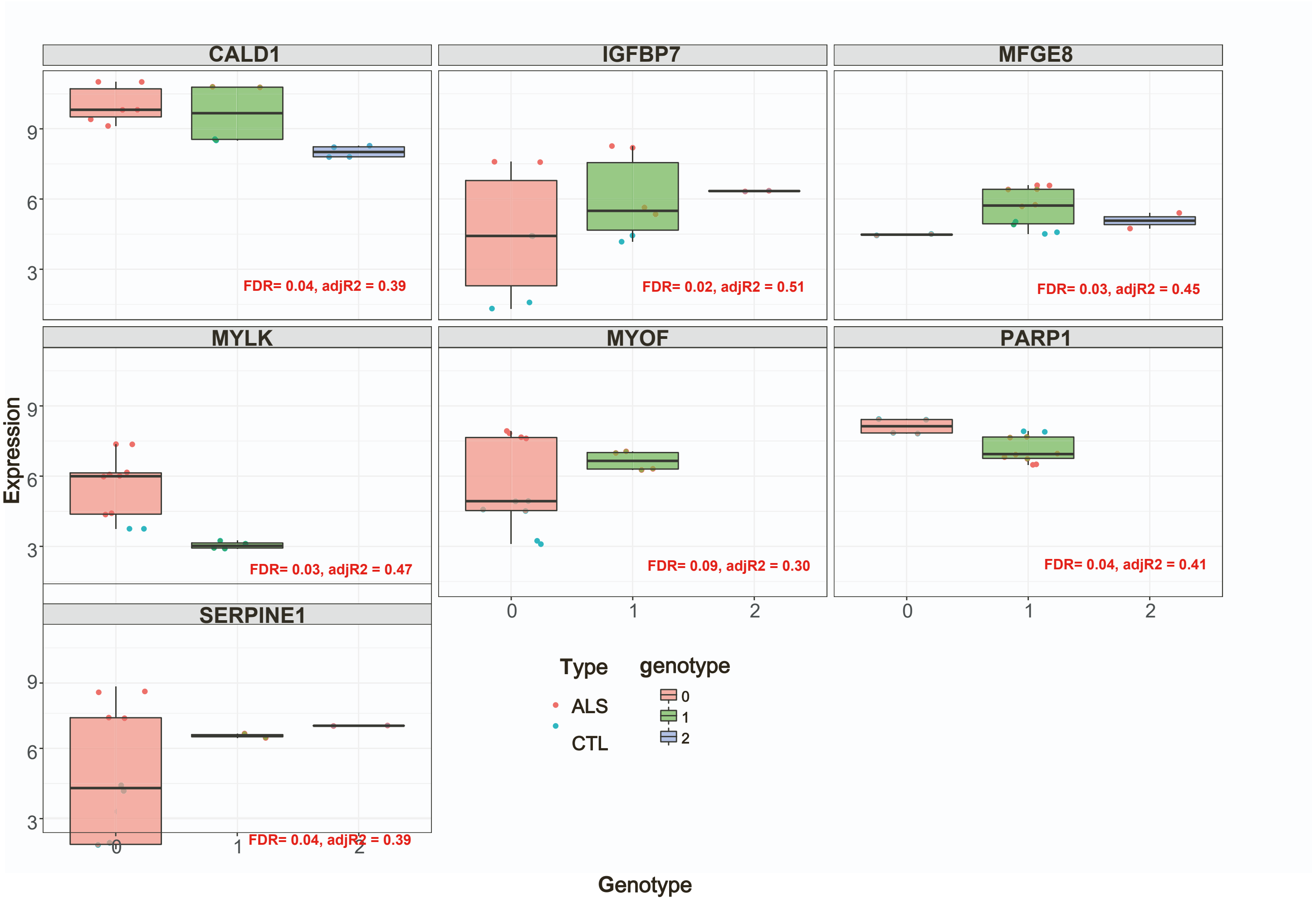

Supplementary Figure 13: eQTLs compared to known brain eQTLs.

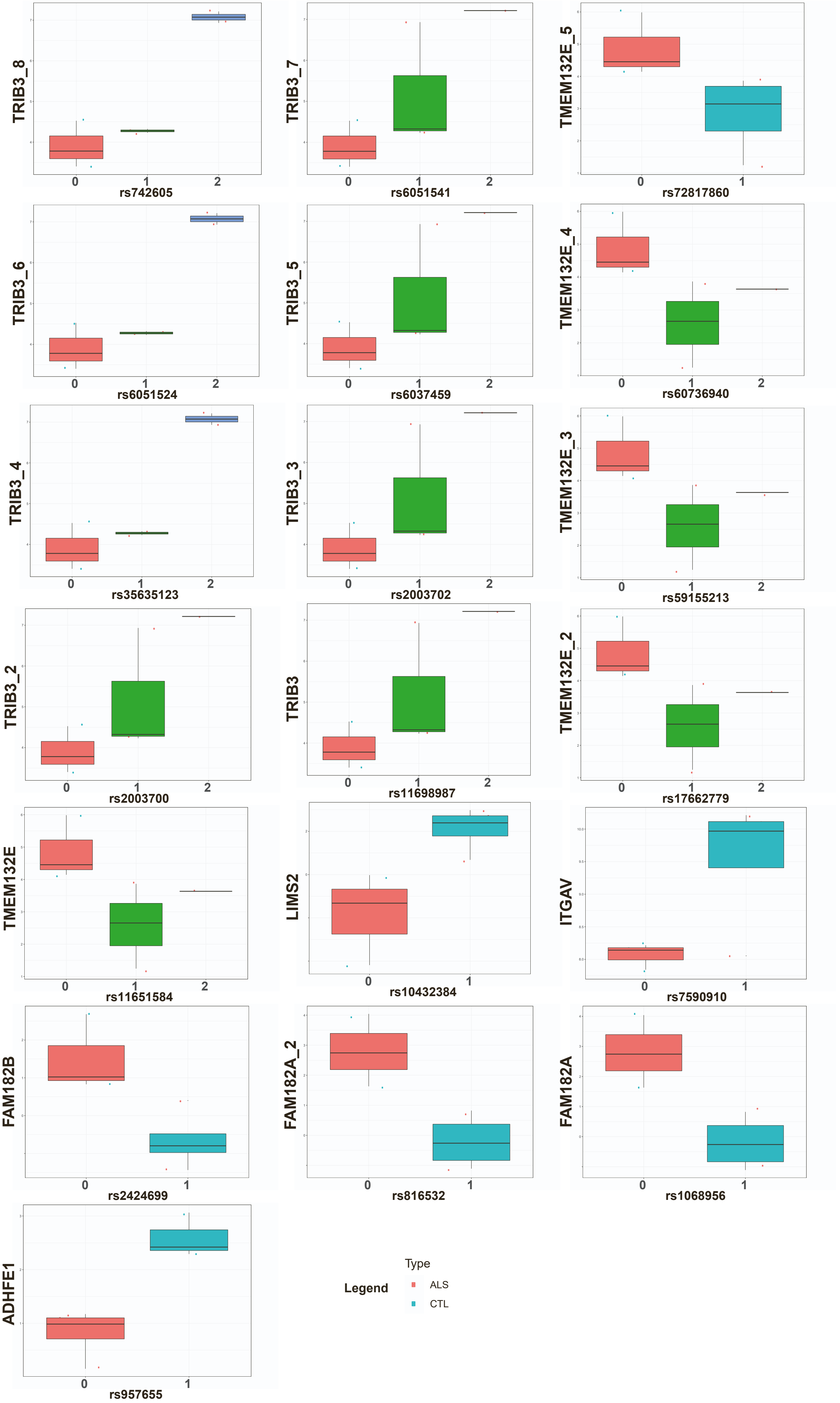

Supplementary Figure 14: Top GO enrichments for each assay.

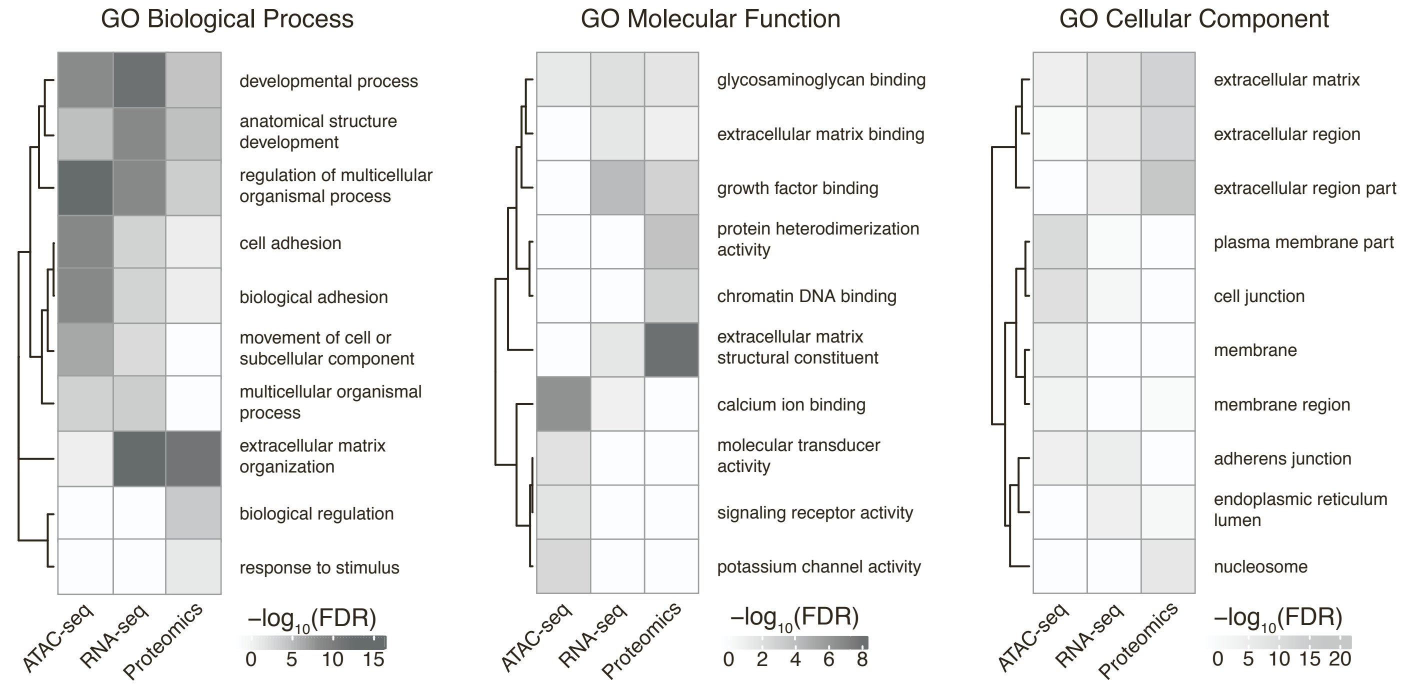

Supplementary Figure 15. SUMO Subnetwork of disease network.

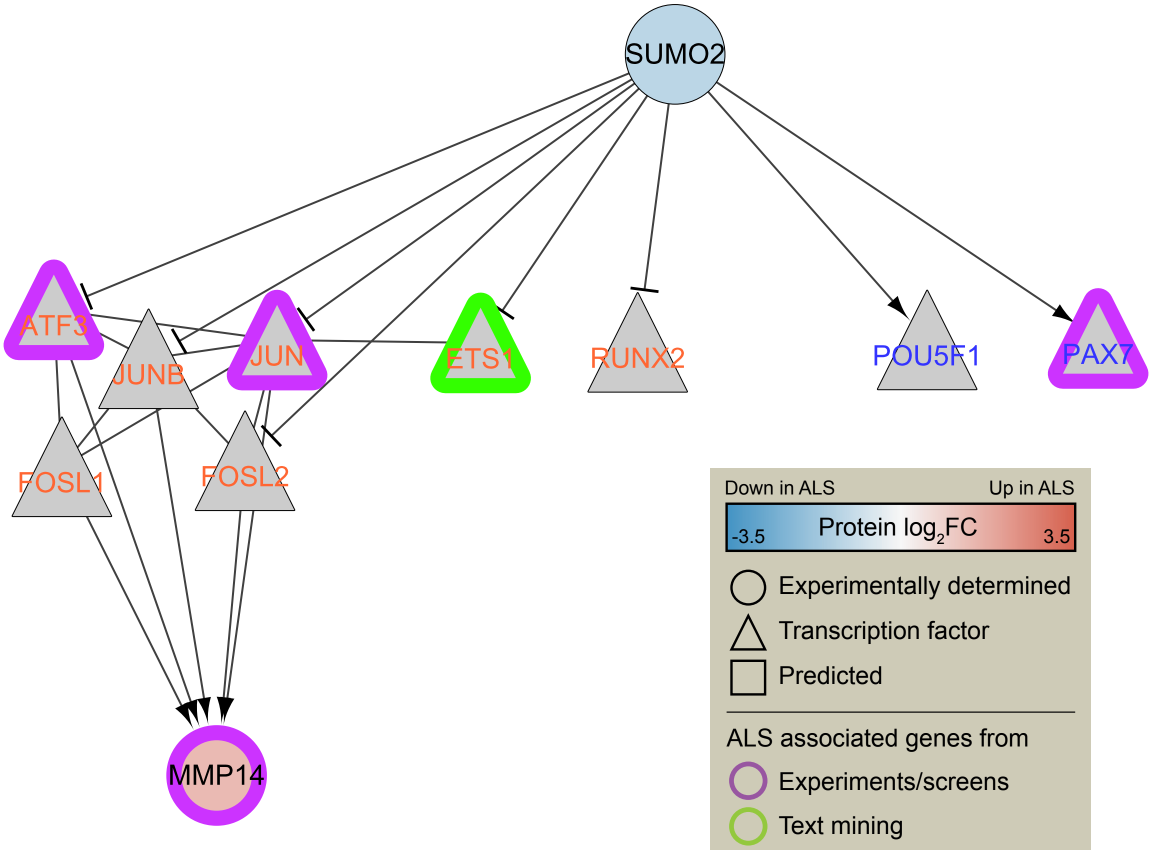

Supplementary Figure 16: Comparison to postmortem cervical spine data.

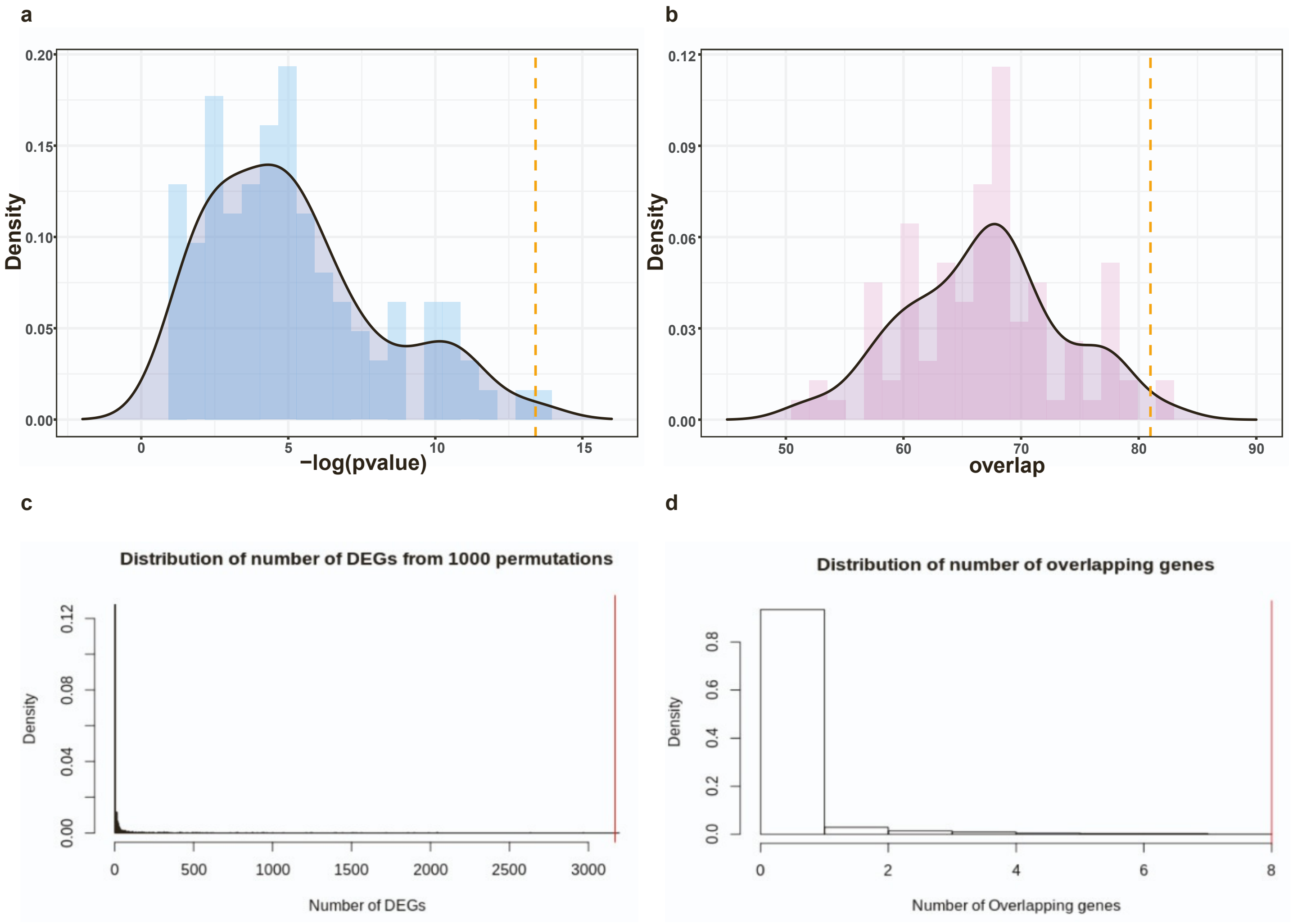

Supplementary Figure 17: Comparison of protein expression and eye phenotype.

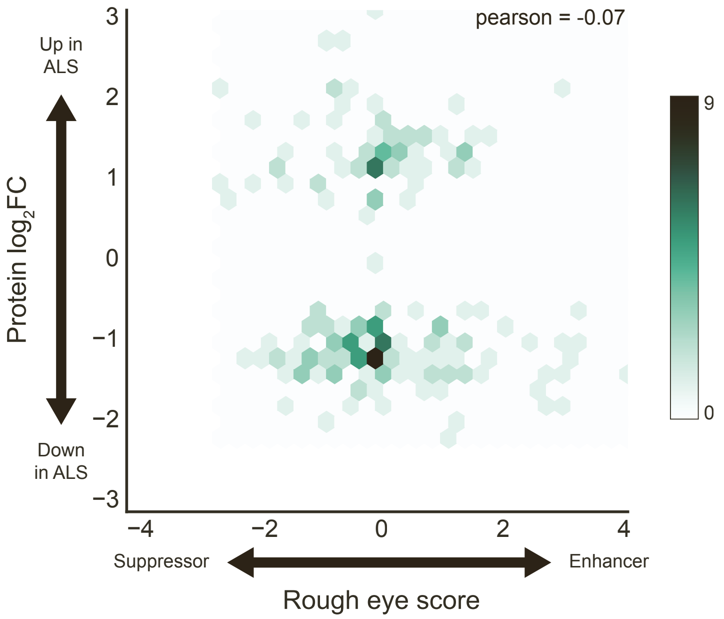

Supplementary Figure 18: Comparison of proteins from the integrated network between two sets of cultured motor neuron experiments.

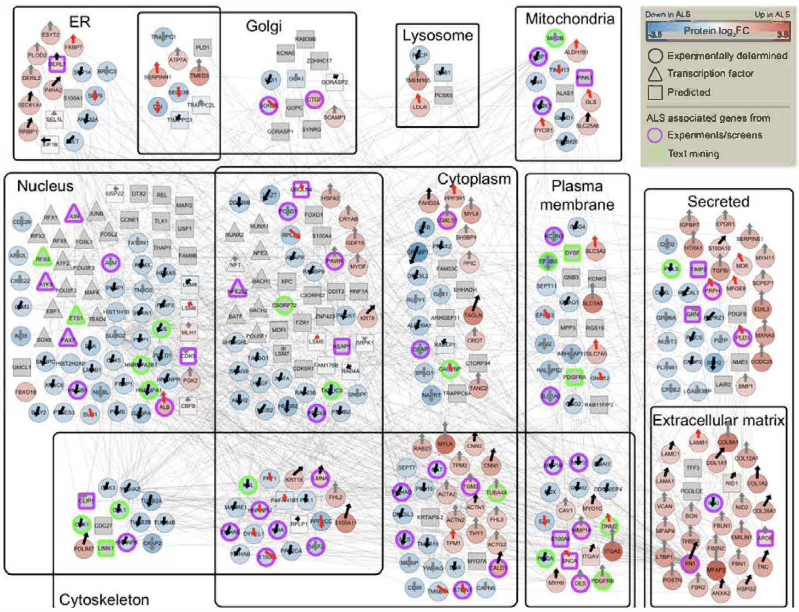

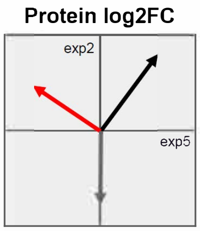

- **Black arrows** indicate nodes that are consistent, with log2FC in the same direction
- **Red arrows** indicate nodes that are inconsistent, with log2FC in opposite directions
- **Gray arrows** indicate nodes that are neither (not detected in both experiments)

- Null hypothesis: equal number of black and red arrows
- 133 black arrows
- 40 red arrows
- Significantly more black than red (pval=1.54E-12)

Supplementary Table 1, iPSC and Differentiation Reagents Stage 1, Related to Figure 1 and STAR methods

Stage 1 (Day 0-Day 6)	Manufacturer	Catalog #	1X Concentration
IMDM	Life Technologies	12440061	47.5%
F12	Life Technologies	11765062	47.5%
NEAA	Life Technologies	11140-50	1%
B27	Life Technologies	17504044	2%
N2	Life Technologies	17502048	1%
Anti/Anti	Life Technologies	15240062	1%
LDN193189	Cayman Chemical	19396	0.2 µM
CHIR99021	Xcess bioscience	M60002	3 µM
SB431542	Cayman Chemical	13031	10 µM

Supplementary Table 2, iPSC and Differentiation Reagents Stage 2 Platedown, Related to Figure 1 and STAR methods

Stage 2 Platedown (Day 6)	Manufacturer	Catalog #	1X Concentration
IMDM	Life Technologies	12440061	47.45%
F12	Life Technologies	11765062	47.45%
NEAA	Life Technologies	11140-50	1%
B27	Life Technologies	17504044	2%
N2	Life Technologies	17502048	1%
Anti/Anti	Life Technologies	15240062	1%
All-trans RA	Stemgent	04-0021	0.1 µM
SAG	Cayman Chemical	11914	1 µM
LDN193189	Cayman Chemical	19396	0.2 µM
CHIR99021	Xcess bioscience	M60002	3 µM
SB431542	Cayman Chemical	13031	10 µM
Rock Inhibitor (Y-27632)	Stemcell Technologies	72308	10 µM

Supplementary Table 3, Stage 2, Differentiation Reagents, Related to Figure 1 and STAR methods

Stage 2 (Day 7-11)	Manufacturer	Catalog #	1X Concentration
IMDM	Life Technologies	12440061	47.5%
F12	Life Technologies	11765062	47.5%
NEAA	Life Technologies	11140-50	1%
B27	Life Technologies	17504044	2%
N2	Life Technologies	17502048	1%
Anti/Anti	Life Technologies	15240062	1%
All-trans RA	Stemgent	04-0021	0.1 μM
SAG	Cayman Chemical	11914	1 μM
LDN193189	Cayman Chemical	19396	0.2 μM
CHIR99021	Xcess bioscience	M60002	3 μM
SB431542	Cayman Chemical	13031	10 μM

Supplementary Table 4, Differentiation Reagents Stage 3, Related to Figure 1 and STAR methods

Stage 3 (Day 12-Day18)	Manufacturer	Catalog #	1X Concentration
IMDM	Life Technologies	12440061	47.5%
F12	Life Technologies	11765062	47.5%
NEAA	Life Technologies	11140-50	1%
B27	Life Technologies	17504044	2%
N2	Life Technologies	17502048	1%
Anti/Anti	Life Technologies	15240062	1%
*SAG	Cayman Chemical	11914	0.1 μM
db-cAMP	Millipore	28745	0.1 μM
All-trans RA	Stemgent	04-0021	0.5 μM
Compound E	Calbiochem	565790	0.1 μM
DAPT	Cayman Chemical	13197	2.5 μM
Ascorbic Acid	Sigma-Aldrich	A4403	200 ng/mL
BDNF (-80)	Peprotech	450-02	10 ng/mL
GDNF (-80)	Peprotech	450-10	10 ng/mL

Supplementary Table 5: iPSC description

C9orf72-AMYOTROPHIC LATERAL SCLEROSIS

iPS Cell Line	iPSC method	Race/ Gender	Age of onset	Age at Samplin	Mutation	Sour ce tissu	iPSC source	Clinical remarks and family history
CS28iALS-C9	Epsiomal	WM	46 YR	47 YR	*C9ORF72 exp.* ~800 G_4C_2	*Fibroblast* WUST	CSMC	Father died of ALS age 70; sister died of ALS age 51; paternal cousins died age 50, 51 of ALS; brother with FTD. Site of onset: Left upper extremity
CS29iALS-C9	Epsiomal	WM	46 YR	47 YR	*C9ORF72 exp.* ~800 G_4C_2	*Fibroblast* WUST	CSMC	Maternal diagnosed with ALS; maternal grandfather diagnosed with dementia and parkinsonism. Site of onset: Left lower extremity
CS30iALS-C9	Epsiomal	WF	51 YR	51 YR	*C9ORF72 exp.* ~70 G_4C_2	*Fibroblast* WUST	CSMC	Mother with FTD maternal uncle and aunt, and two cousins died of ALS. Site of onset: Bulbar
CS52iALS-C9	Epsiomal	WM	45 YR	48 YR	*C9ORF72 exp.* ~800 G_4C_2	*Fibroblast* WUST	CSMC	Father died age 63 with dementia; paternal grandmother died of bulbar onset ALS. Site of onset: Left upper extremity

UNAFFECTED CONTROLS

iPS Cell Line	iPSC method	Race/ Gender	Age at Sampling	Diagnosis	Group/ Gene	Sour ce tissu	iPSC source	
CS00iCTR-21n	Epsiomal	AAM	6 YR	Clinically normal	N/A	*Fibroblast*	CSMC	Apparently healthy non-fetal tissue
CS83iCTR-33n	Epsiomal	WF	21 YR	Clinically normal	HD negative	*Fibroblast*	CSMC	Asymptomatic; HD Gene-negative, Apparently healthy non-fetal tissue
CS25iCTR-18n	Epsiomal	WM	76 YR	Clinically normal	HD negative	*Fibroblast*	CSMC	Asymptomatic; HD Gene-negative. Apparently healthy non-fetal tissue

Table S5, Related to Figure 1: **Clinical and reprogramming details for the ALS-C9 and control iPSC lines used in the NeuroLINCS study.** W: White; AA: African American; M: Male; F: Female; CS: Cedars-Sinai; HD: Huntington Disease.

Supplementary Table 6: Variants in iPSCs

Variant Type	Total Variants
Total variants in the 3 control iPSC lines	9,197,462
Exonic functional	57,910
Rare Exonic functional < 1% frequent or novel (no frequency information)	12,898
Regulatory	4,947,479
Total variants in iPSC lines with C9ORF72 mutation	8,818,235
Exonic functional	55,815
Rare Exonic functional < 1% frequent or novel (no frequency information)	8,225
Regulatory C9ORF72	4,740,665

Table S6, Related to STAR methods: Summary Table of all variants in iPSC lines from three healthy volunteers and four individuals with ALS due to C9ORF72 mutation. The total number of variants that are exonic functional are reported. These are nonsynonymous variants which include missense, splicing. frameshift, non-frameshift, stop-gain and start loss variants only. For regulatory variants, we filtered for variants that are in intergenic and regulatory regions. We report the variant as found next to the closest gene, these will be either in the 5’ or 3’ UTR, intronic, upstream and downstream up to 4 KBs from the start and stop of a gene.

Supplementary Table 7: ALS-Associated Variants

Specific ALS Associated Variants Found (ALSoD database)	Control Line	C9ORF72 Line
OPTN c.293T>A:p.M98K:(16Exons):exon5:missense (3.0% frequent)	CS83iCTR-33n1	CS28iALS-C9n2
ALS2 c.280A>G:p.I94V(4Exons):exon4:missense (2.1% frequent)	-	CS52iALS-C9n6
DIAPH3 c.974C>T:p.P325L:(16Exons):exon10:missense (3.6 % frequent)	CS83iCTR-33n1	-

Table S7, Related to STAR methods: **Summary Table of all ALS variants in the control and C9ORF72 lines.** ALS specific variants were found in one of the CS83iCTR-33n1 and 2 of the C9ORF72 lines.

Supplementary Table 8: Functional Variants

Variants that shared across _ and are in _	Total Variants	Variants that overlap with RNA Seq data	Variants that overlap with ATAC Seq data	Non-synonymous (includes Start-loss (STL)	Frame Shift (FS)	Splicing (S)	Stop-gain (SG)	Stop-loss (SPL)
3 controls and all C9ORF72 (cases) lines	98,897	4,908	847	29,052	736	872	365	43
4 cases, 0 controls	206	16	1	82	2	1	0	0
4 cases, 1 control	910	62	8	312	4	11	8	1
4 cases, 2 controls	2,774	186	22	1,047	16	37	8	2
3 cases, 0 controls	799	49	7	290	9	11	4	1
3 cases, 1 control	2,259	155	33	828	11	18	5	2
2 cases, 0 controls	2,784	195	37	1,021	37	17	17	0
1 case, 3 controls	638	52	4	222	4	11	0	0
0 case, 3 controls	218	7	4	96	1	2	0	1
0 case, 2 controls	1,405	79	13	509	11	20	10	3
Total enriched in cases or controls	11,993	801	129	4,407	95	128	52	10

Table S8, Related to STAR methods: Summary table showing the number of functional variants that are shared across different subsets of cases and controls. The top row indicates the variant type. For example, the second row from the top lists the total number of exonic variants that are shared across all control and C9ORF72 lines while the third row from the top lists the total number of variants that are shared across only cases but are not found within any controls. Each row lists the subset of lines for which variants are shared.
